# Stochastic optimization for minimizing operational costs in smart hybrid energy networks considering electric vehicle

**DOI:** 10.1371/journal.pone.0323491

**Published:** 2025-06-09

**Authors:** Nouman Qamar, Mohammed Alqahtani, Muhammad Rehan, Ijaz Ahmed, Muhammad Khalid

**Affiliations:** 1 Electrical Engineering Department, University of Engineering and Technology, Punjab, Pakistan; 2 Department of Industrial Engineering, King Khalid University, Abha, Saudi Arabia; 3 Electrical Engineering Department, King Fahd University of Petroleum & Minerals (KFUPM), Dhahran, Saudi Arabia; 4 Interdisciplinary Research Center for Sustainable Energy Systems (IRC-SES), KFUPM, Dhahran 31261, Saudi Arabia; Aalto University, FINLAND

## Abstract

The residential energy hub (REH) effectively satisfies power demands, but the incorporation of renewable energy sources (RES) and the increasing use of plug-in hybrid electric vehicles (PHEVs), with their unpredictable nature, complicates its optimal functionality and challenges the accurate modeling and optimization of REH. This work proposed a stochastic model for REH using mixed integer linear programming (MILP) to optimally handle the associated uncertainties of RES and PEHVs, which was then solved using GAMS software. Four case studies with varying conditions were conducted to verify the performance of the proposed scheme, and the results indicate that the approach is superior in optimally handling the system’s associated limitations. These limitations include the intermittency and variability of RES and the uncertainties associated with PHEVs, such as arrival time, travel distance, and departure time. Additionally, this work introduces a smart charging mechanism that charges and discharges PHEVs economically, both in terms of cost and reliability. The results indicate that incorporating a smart charging mechanism decreases the total operating cost of smart REH by 2.59% while maintaining the comfort level of the consumer and increasing the reliability of the overall system. Finally, smart REH adopts a demand response program (DRP), which further reduces the operational cost by 3.7%. Furthermore, the proposed approach demonstrates a significant reduction in operating costs and an improvement in the reliability of the smart REH.

## 1 Introduction

The global energy demand for the residential sector is increasing rapidly. Anticipated growth in residential sector demand is likely to result in more reliance on renewable energy sources (RES) and novel energy storage systems (ESSs) [1, 2]. Home energy management systems (HEMS) now simultaneously meet the electrical, cooling, and heating requirements of residential buildings due to the emergence of the energy hub (EH) concept and improved energy form conversion efficiency [3, 4]. Residential facilities primarily consume energy due to their electrical, heating, and cooling requirements. However, optimizing the functioning of these systems presents a challenge, as it is crucial for enhancing the overall efficiency of both electricity and gas grids [5, 6]. This highlights the critical research gap in the development of advanced solutions to improve the integration and control of energy resources in residential buildings [7, 8].

The EH concept, which can handle and convert different forms of energy, is gaining popularity in research for the optimization of HEMS [9, 10]. Adopting different energy management techniques from EH can optimize the energy purchasing cost of residential buildings [11]. Residential buildings are increasingly using RES to reduce energy costs and reduce their reliance on fossil fuels [12]. It is crucial to integrate RES into EH systems to enhance their contribution to the building’s energy consumption. However, given their intermittent nature, accurately predicting and optimizing their share in meeting the demands of REH is a challenging task [13]. The use of ESS can mitigate the uncertainty associated with the RES, increasing system reliability and lowering operational costs [14–16]. The use of an electric vehicle (EV) in place of a conventional vehicle is increasing rapidly for a cleaner environment. The use of EVs in residential buildings increases the burden on the electrical network, but proper utilization of EV batteries can improve energy cost optimization [17, 18]. The availability of EVs in EH is dependent on the unpredictable departure and arrival times of the EV’s owner [19]. This limits the use of EVs as a complete storage device for energy. Additionally, EVs require smart charging for their daily travel routine, as improper charging could burden the REH and reduce its efficiency [20, 21]. The proper size and smart charging are required to properly and optimally integrate the EV with the REH [22, 23].

Despite extensive research on EV integration in EH, it lacks the complete stochastic modeling of all the uncertain components necessary for precise and accurate results in the shortest amount of time [24, 25]. Therefore, there is a pressing need to propose a fast and accurate technique for the optimal operation of REH with RES and PHEV [26, 27]. Developing a new technique based on scenario generation and reduction methods, in conjunction with the smart charging of PHEVs, would provide a suitable solution for the optimal operation of REH, ensuring speed and accuracy. The uncertainties of RES and PHEV availability pose an adverse effect on the operation of REH, but by identifying and negating their negative impact, optimal operation of REH can be achieved [28, 29]. Scholars in [30], optimal energy scheduling of REH in the presence of storages is discussed along with demand side management (DSM). A two-stage multi-criteria optimization technique is presented that improves the storage lifetime of storage devices in the first step and optimizes the REH in the second step [31]. Although this technique increases the life of energy storage devices, it lacks the inclusion of intermittent RES and uncertain PHEV. The study [32] proposes a solution to the scheduling problem of smart REH (SREH), which considers various uncertain parameters such as solar radiation, energy demands, and electricity costs, using the Monte Carlo simulation technique. However, it ignores the integration of PHEV into REH. In [33], authors introduced an IoT-enabled approach for optimizing multi-EH, including RES and PHEV, that addresses the uncertain nature of RES in a correlated environment. In [34], we present a deep learning-based optimal scheduling of a virtual energy hub in the presence of plug-in hybrid CNG (compressed natural gas) EVs.

Some references have focused on the handling of uncertainties associated with RES through probabilistic or stochastic modeling [35]. The authors in [36] developed a probabilistic operation strategy for the optimal operation of EH with different energy converters by proposing various innovative uncertainty modeling techniques. In [37], the authors propose a probabilistic correlation of RES in EH to maximize its profit, based on the game theory approach. In study [38], the authors discuss a stochastic framework for energy management for SEH that utilizes a false data detection scheme. Wang *et al*. [39], presents a new stochastic optimal SREH management system that seeks to reduce energy usage, GHG emissions, and energy expenditures by determining the optimal power output for desert regions. Abdul *et al*. [40], researchers investigate a two-stage stochastic optimization of EH that considers different types of storage in the presence of RES and EVs. The first stage focuses on determining the optimum size and placement of each EH, while the second stage addresses the optimum charging and discharging of each component and EV. The results show that smart charging and discharging of EVs can reduce the total operating cost by approximately 5.5%. Some authors have applied the concept of a microgrid as a decentralized EH by integrating and managing different energy sources. Their dynamic model of control systems enables efficient, sustainable, and resilient energy management, allowing it to operate independently from the main grid while providing reliable power [41, 42]. Shokri *et al*. [43], introduced a novel concept for the optimal operation of smart cities as energy hubs. Their EH model incorporates heat pumps, fuel cells, microturbines, ESSs, desalination units, RES, and EVs. They applied a biogeography-based optimization algorithm to an enhanced IEEE 33-bus test system to demonstrate its effectiveness. The results show a significant decrease in costs and environmental pollution.

Many scholars have successfully integrated the RES to REH, and some have also effectively managed the uncertainty. Table 1 reveals the major novelty of proposed work in terms of modeling and associated constraints and research gap in the stochastic modeling and integration of PHEV, along with its smart charging and DSM. While some researchers have successfully integrated the PHEV into the REH and addressed its uncertainty, the absence of a smart charging mechanism hinders its full optimization. Therefore, a comprehensive modeling of REH is necessary, one that not only addresses the uncertain behavior of RES and PHEV parameters, but also incorporates a smart charging/discharging mechanism for the PHEV in conjunction with the DSM system.

**Table 1:** Comparison of the proposed SREH with relevant literature.

References	Uncertainty modeling of RES	DSM	PHEV	Uncertainty modeling of PHEV	Smart charging of PHEV	
[44]	✓	✓	✓	×	×	×
[45]	✓	✓	✓	×	×	×
[46]	✓	✓	✓	✓	×	×
[46]	✓	✓	×	×	×	×
[47]	✓	✓	✓	×	×	×
[48]	✓	✓	✓	✓	×	×
[49]	✓	✓	×	✓	✓	×
[50]	✓	✓	✓	×	×	×
[51]	✓	✓	✓	✓	×	×
[52]	✓	×	×	✓	✓	✓
[53]	✓	×	×	✓	✓	✓
[54]	✓	✓	×	✓	✓	×
[55]	✓	✓	✓	✓	×	×
[56]	✓	×	×	✓	×	×
[57]	✓	✓	✓	✓	×	×
[58]	✓	✓	✓	×	×	×
[59]	✓	✓	✓	✓	✓	×
[60]	✓	✓	✓	×	×	×
Proposed Model	✓	✓	✓	✓	✓	✓

### 1.1 Contribution and novelties

The following outline highlights the key contributions of this research to the field of optimal stochastic operation of REH.

1. In contrast to earlier research in [61–64], a more complex and practical model has been developed that takes into account the uncertain charging limits of both RES and PHEVs, as well as factors like arrival time, travel distance, smart charging/discharging, and departure time. The developed model is more reliable and achieved optimal economic aspects in terms of cost and reliability.
2. The proposed approach signifies a notable improvement over prior research works by concurrently tackling the uncertainties linked to RES and PHEVs, resulting in enhanced accuracy and reliability of outcomes. The simultaneous examination of uncertainty improves the model’s robustness and complexity, distinguishing it from previous models that addressed either RES [40, 56] or PHEV challenges [52, 65] individually.
3. The suggested methodology is novel relative to previous works [17, 23, 66], since it incorporates an intelligent decision-making process for the charging and discharging of PHEVs into the REH scheduling to enhance its optimal performance.
4. This study presents a broader strategy by concurrently applying DSM approaches to electrical, heating, and cooling loads, in contrast to prior studies such as [67, 68], which concentrated exclusively on dispatch models for electric loads. The incorporation of heating and cooling systems substantially improves the model’s relevance, making it more adept at handling the intricate and varied load requirements in contemporary energy systems.
5. The proposed work conducted a comparative study of all contributions (see Table 1 references [32-48]) and [69] to ascertain the relative efficacy of each comparative feature (DSM, smart charging of PHEVs, and the unpredictable nature of RES).
6. Unlike the works in [9, 70–72], which use distributed methods to combine RES through RETScreen, the proposed framework is based on MILP and GAMS software, which gives better outcomes and is simple to use for complex energy delivery problems.

The proposed method introduces significant improvements over existing methods, such as smart charging strategies for PHEVs that dynamically adjust based on real-time demand, electricity prices, and the availability of RES. This feature enhances the efficiency of the SREH by optimizing the charging/discharging cycles of PHEVs in addition to the DSM. The combined stochastic modeling of solar irradiance, wind speed, and PHEV usage patterns provides more accurate forecasting and optimization under real-world uncertainties, which is crucial for optimizing EHs in smart grids [73]. Another novel aspect of our technique is the identification of the feasible operating region for the SREH components based on their constraints. Previous methods mainly focus on the isolated optimization of PHEV charging/discharging and RES without considering real-time interactions between them. The proposed method improves on this approach by simultaneously optimizing the charging/discharging behavior of PHEVs and the energy management of the REH while also incorporating DSM strategies. This results in a more comprehensive and adaptive solution for energy management in modern grids.

The article continues with the following sections: Section 2 describes the problem description. Section 3 formulates the mathematical modeling of the problem. Section 4 describes the methodology to solve the problem. Section 5 presents simulation and case studies. The results are discussed in section 6. Finally, conclusions are drawn in section 7.

## 2 Problem description

### 2.1 REH architecture

**Fig 1:**
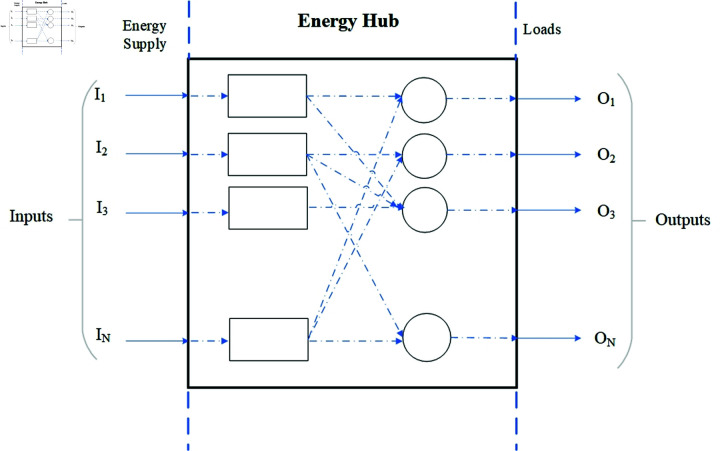
Energy hub architecture.

The concept of EH has evolved to serve as an interface between diverse input energy carriers and output requirements, as noted by Najafi *et al*. [74]. The EH facilitates the conversion of one kind of energy into another as needed to satisfy specific demands. The fundamental model of EH is depicted in Fig. 1, with inputs denoted as I and outputs denoted as O. The inputs and outputs are connected by (1).

I=C*O (1)

where *C* represents the coupling factor between the inputs and outputs. The multiple inputs and outputs of EH make *C* a matrix, as defined in (2) [75], which denotes the topology, dispatch factor, and converter efficiency of EH.

$$\left[\begin{array}{c} \begin{array}{c} I_{1}\\ I_{2}\\ \vdots \\ I_{i}\\ \vdots \\ I_{N} \end{array} \end{array}]=[\begin{array}{c} \begin{array}{cccc} C_{11} & C_{12} & \cdots & C_{1N}\\ C_{21} & C_{22} & \cdots & C_{2N}\\ \vdots & \vdots & \ddots & \vdots \\ C_{i1} & C_{i2} & \cdots & C_{iN}\\ \vdots & \vdots & \ddots & \vdots \\ C_{N1} & C_{N2} & \cdots & C_{NN} \end{array} \end{array}][\begin{array}{c} \begin{array}{c} O_{1}\\ O_{2}\\ \vdots \\ O_{i}\\ \vdots \\ O_{N} \end{array} \end{array}\right]$$ (2)

**Fig 2:**
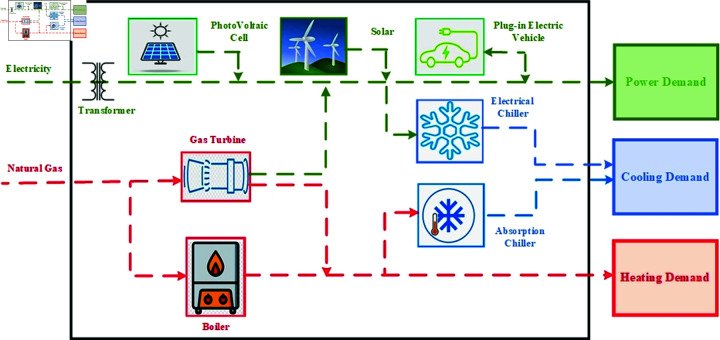
Conceptual framework of REH under study.

The idea of EH can be used on different levels, like residential EH, commercial EH, industrial EH, agricultural EH, etc. [76]. This paper applies the concept of EH to REH, which not only incorporates the basic components of EH but also integrates PHEV. The basic architecture of REH is shown in Fig. 2. This work incorporate a smart features such as DSM, smart charging of PHEV, accurate modeling of RES, and demands to make REH smarter such as SREH.

### 2.2 Handling procedure for uncertainties

The handling of uncertainties related to customer-owned RES and demands of REH is a challenge for the optimum scheduling of SREH [77]. Also, the uncertain behavior of PHEV owners, like time of departure and time of arrival, etc., must be properly handled to accurately model the charging/discharging of PHEV [78]. The optimization of REH is not possible without handling these uncertainties. Deterministic modeling cannot adequately handle the uncertainties related to solar irradiance and wind speed for solar and wind power production. To handle these uncertainties, either probabilistic or stochastic modeling is required, [79, 80]. In this work, probability density functions (PDFs) have been applied in order to consider the random behavior of PHEVs, wind speed, and solar irradiance. The normal PDF is used to model uncertainties related to PHEVs, the Weibull PDF is used for wind speed, and the beta PDF is used to model solar irradiance. The powers generated from solar and wind are calculated using (3) and (4), respectively.

$$P_{\text{PV}}^{e}(S_{i}(t,s))={\text{PV}}_{\text{array}}\cdot {\text{df}}_{\text{PV}}\cdot S_{i,\text{STC}}\cdot \left(1+\alpha_{p}\left(T_{c}-T_{c,\text{STC}}\right)\right)$$ (3)

where $PV_{\text{array}}$ is the size of PV panels, $df_{\text{PV}}$ is the derating factor of PV panels, $S_{i,\text{STC}}$ is the solar irradiance at standard test conditions, $\alpha_{p}$ is the coefficient of temperature, and $T_{c,\text{STC}}$ is the PV cell temperature under standard test conditions.

$$P_{\text{wind}}^{e}(\omega (t,s))=\{\begin{array}{cc} P_{\text{out}}^{w} & \text{if }\omega_{r}^{w}\leq \omega_{t}\leq \omega_{\text{ocut}}\\ \frac{P_{\text{out}}^{w}(\omega_{t}-\omega_{i}^{\text{cut}})}{\left(\omega_{r}^{w}-\omega_{i}^{\text{cut}}\right)} & \text{if }\omega_{i}^{\text{cut}}\leq \omega_{t}\leq \omega_{r}^{w}\\ 0 & \text{if }\omega_{t}\leq \omega_{i}^{\text{cut}}\text{ or }\omega_{t}\geq \omega_{\text{ocut}} \end{array}$$ (4)

where $P_{\text{out}}^{w}$ is the power output of wind turbine (WT), $\omega_{r}^{w}$ is the rated speed of WT, $\omega_{i}^{\text{cut}}$ is the cut-in speed of WT, and $\omega_{o}^{\text{cut}}$ is the cut-out speed of WT.

**Fig 3:**
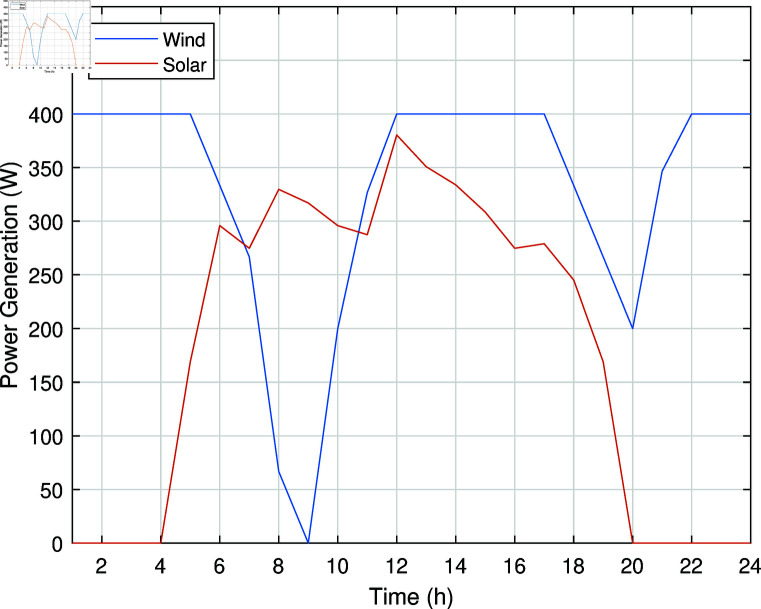
Hourly power generation of solar and wind.

The hourly power generation of solar and wind is shown in Fig. 3.

**Fig 4:**
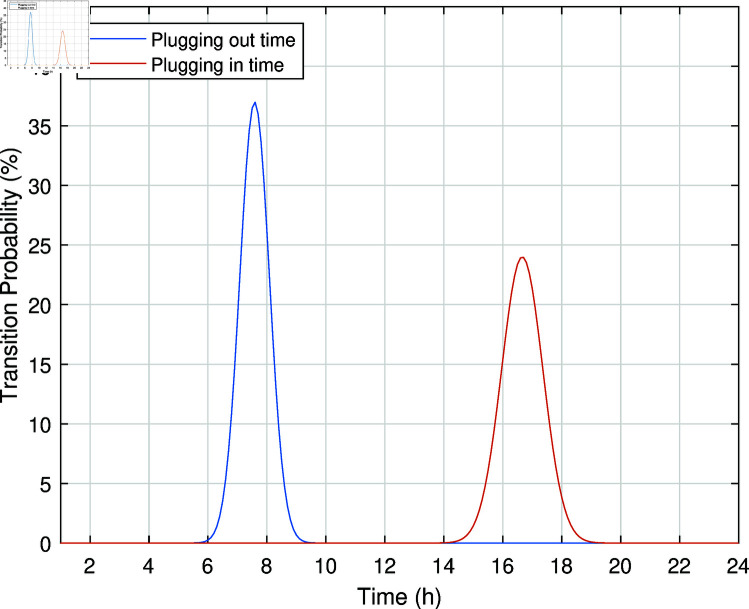
Probability of PHEV’s time of departure and time of arrival.

This work handled the uncertainties related to PHEV, such as the time of arrival, departure, and traveling distance, in addition to the uncertainties related to RES. The statistical historical data of PHEV drivers is shown in Fig. 4 [81]. The mean departure time is 7:35 with a standard deviation (SD) of 0.6 hours, while the mean arrival time is 16:40 with an SD of 0.9 hours.

## 3 Mathematical modeling

**Fig 5:**
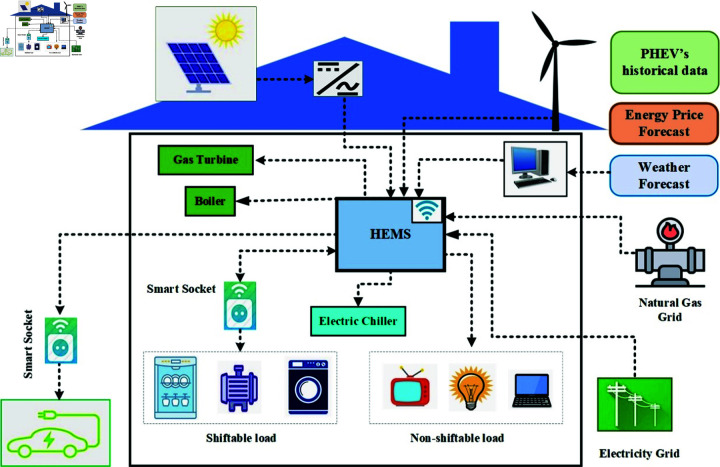
Proposed model of energy management for SREH.

The proposed structure for SREH, which is optimized by smart HEMS (SHEMS), is presented in Fig. 5. The REH includes various components, such as CHP, which converts gas energy into heat and electrical energy; RES, which produces electrical energy; electric chillers and absorption chillers that fulfill cooling demand; and a boiler that generates heat using natural gas. To better fulfill REH’s load demand, electrical loads are further divided into two types: shiftable and non-shiftable. Shiftable loads are flexible and can operate at any time of the day. These include appliances such as dishwashers, washing machines, and water pumps. On the other hand, one must operate non-shiftable loads as needed, without the flexibility to shift them to a different time of day. These include household appliances such as lighting and heating/cooling systems.

### 3.1 Objective function

The total operational cost of SREH is calculated using the objective function given in (5).

$$\text{Cost}=\sum_{t=1}^{24}\sum_{s=1}^{10}\psi_{s}[\lambda_{e}(t,s)P_{\text{Grid}}^{E}(t,s)+\lambda_{g}(t,s)P_{\text{in}}^{\text{GAS}}(t,s)+\lambda_{w}P_{\text{wind}}^{e}(t,s)+\lambda_{S}P_{\text{PV-final}}^{e}(t,s)]$$ (5)

where $\psi_{s}$ is the probability of scenario *s*, $\lambda_{e}$ is the cost of electricity purchased from the grid, $P_{\text{Grid}}^{E}$ is the electrical power purchased from the grid, $\lambda_{g}$ is the cost of gas purchased from the grid, $P_{\text{in}}^{\text{GAS}}$ is the gas purchased from the grid, $\lambda_{w}$ is the operational cost of WT, $P_{\text{wind}}^{e}$ is the electrical power produced from the WT, $\lambda_{S}$ is the operational cost of PV panels, and $P_{\text{PV-final}}^{e}$ is the electrical power produced from the PV panels.

### 3.2 Electrical components

The electrical portion of SREH received electrical power from the grid and, through a transformer, converted high-potential voltage to low-potential voltage to feed it to the loads and other components of SREH. The equation for the conversion of high voltage to low voltage is given by (6).

$$P_{\text{in}}^{E}(t,s)=\eta_{\text{Trans}}^{E}\cdot P_{\text{Grid}}^{E}(t,s)$$ (6)

where $P_{\text{in}}^{E}$ is the electrical power output of the transformer, $\eta_{\text{Trans}}^{E}$ is the efficiency of the transformer, and $P_{\text{Grid}}^{E}$ is the purchased electrical power from the grid.

Using an inverter, the solar power generated from PVs is converted from DC to AC to feed it to the electrical portion of SREH and is denoted by (7).

$$P_{\text{pv-final}}^{e}(t,s)=\eta_{\text{DC/AC}}^{\text{pv}}\cdot P_{\text{pv}}^{e}(t,s)$$ (7)

where $P_{\text{pv-final}}^{e}$ is the final production of PV panels in AC, $\eta_{\text{DC/AC}}^{\text{pv}}$ is the efficiency of the inverter to convert DC to AC, and $P_{\text{pv}}^{e}$ is the production of DC electrical power of PV panels.

The conversion of gas to electrical power using a combined heat plant (CHP) unit is denoted by (8).

$$P_{\text{CHP}}^{e}(t,s)=\eta_{\text{CHP}}^{e}\cdot P_{\text{CHP}}^{\text{gas}}(t,s)$$ (8)

where $P_{\text{CHP}}^{e}$ is the electrical power produced by CHP, $\eta_{\text{CHP}}^{e}$ is the efficiency of CHP to produce electrical power from gas, and $P_{\text{CHP}}^{\text{gas}}$ is the power consumed by CHP.

The minimum and the maximum limits of buying electrical power from the grid are given by (9).

$$0\leq P_{\text{Grid}}^{E}(t,s)\leq P_{\text{Gridmax}}^{E}$$ (9)

where $P_{\text{Gridmax}}^{E}$ is the maximum power that can be purchased from the grid at any interval of time.

The permissible limits for CHP and electric chillers are given by Eqs. 10 and (11) respectively.

$$0\leq P_{\text{CHP}}^{e}(t,s)\leq P_{\text{CHP max}}^{e}$$ (10)

$$0\leq P_{\text{chill}}^{e}(t,s)\leq P_{\text{chill max}}^{e}$$ (11)

where $P_{\text{CHP max}}^{e}$ and $P_{\text{chill max}}^{e}$ are the maximum electrical powers that can be produced from CHP and electric chillers, respectively.

### 3.3 Heating components

A boiler and CHP use the gas input to the SREH to generate electrical and heat power. It is denoted by (12).

$$P_{\text{in}}^{\text{GAS}}(t,s)=P_{\text{CHP}}^{\text{gas}}(t,s)+P_{\text{Boil}}^{\text{gas}}(t,s)$$ (12)

where $P_{\text{CHP}}^{\text{gas}}$ is the gas power consumed by CHP to produce heat and electrical power, and $P_{\text{Boil}}^{\text{gas}}$ is the gas consumed by the boiler to produce heating power.

The conversion of gas to heating power through CHP is denoted by (13).

$$P_{{\text{CHP}}_{h}}^{H}(t,s)=\eta_{{\text{CHP}}_{h}}^{H}\cdot P_{\text{CHP}}^{\text{gas}}(t,s)$$ (13)

where $P_{{\text{CHP}}_{h}}^{H}$ is the heating power generated by CHP, and $\eta_{{\text{CHP}}_{h}}^{H}$ is the heat generation efficiency of CHP.

The gas is also used by the boiler to produce heating power. The conversion of gas to heating power through the boiler is given in (14).

$$P_{\text{Boil}}^{H}(t,s)=\eta_{\text{Boil}}^{H}\cdot P_{\text{Boil}}^{\text{gas}}(t,s)$$ (14)

where $P_{\text{Boil}}^{H}$ denotes the heating power by boiler, and $\eta_{\text{Boil}}^{H}$ is the heat generation efficiency of the boiler.

The minimum and the maximum limits of buying gas from grid are given by (15).

$$0\leq P_{\text{in}}^{\text{GAS}}(t,s)\leq P_{\text{in max}}^{\text{GAS}}$$ (15)

where $P_{\text{in max}}^{\text{GAS}}$ is the maximum gas bought from gas grid in an interval of time.

The allowable limit for the boiler is given by (16).

$$0\leq P_{\text{Boil}}^{H}(t,s)\leq P_{\text{Boil max}}^{H}$$ (16)

where $P_{\text{Boil max}}^{H}$ is the maximum limit of the boiler to produce heating power in any interval of time.

### 3.4 Cooling components

To meet some of the cooling load, an electric chiller converts electric power into cooling power. The conversion of electrical power to cooling power through an electric chiller is given by (17).

$$P_{\text{ec}}^{C}(t,s)=\eta_{\text{chill}}^{C}\cdot P_{\text{chill}}^{e}(t,s)$$ (17)

where $P_{\text{ec}}^{C}$ is the cooling power by the electric chiller, $\eta_{\text{chill}}^{C}$ is the electric chiller efficiency to convert electric power into cooling power, and $P_{\text{chill}}^{e}$ represents an electrical power used by the chiller for a cooling purpose.

The absorption chiller converts the heating power into cooling power. The conversion of heating power into cooling power through an absorption chiller is given by (18).

$$P_{\text{ac}}^{C}(t,s)=\eta_{\text{ac}}^{C}\cdot P_{\text{ac}}^{H}(t,s)$$ (18)

where $P_{\text{ac}}^{C}$ is the cooling power produced by the absorption chiller, $\eta_{\text{ac}}^{C}$ is the absorption chiller efficiency to convert electric power into cooling power, and $P_{\text{ac}}^{H}$ leads to the heating power used by absorption chiller.

The allowable limit for the absorption chiller is given by (19).

$$0\leq P_{\text{ac}}^{C}(t,s)\leq P_{\text{ac max}}^{C}$$ (19)

where $P_{\text{ac max}}^{C}$ is the maximum cooling power produced by the absorption chiller at an interval of time.

### 3.5 Load balancing constraints

The electric, heating, and cooling requirements of the SREH must be satisfied at each time interval. The constraints for these loads are specified by Eqs. 20 to (22), respectively.

$${\text{Load}}^{E}(t,s)+P_{\text{chill}}^{e}(t,s)+P_{\text{Ch}}^{\text{EV}}(t,s)+P_{\text{high.DR}}^{E}(t,s)=P_{\text{in}}^{E}(t,s)+P_{\text{CHP}}^{e}(t,s)+\;P_{\text{wind}}^{e}(t,s)+P_{\text{pv-final}}^{e}(t,s)+P_{\text{low.DR}}^{E}(t,s)+P_{\text{Dis}}^{\text{EV}}(t,s)$$ (20)

where ${\text{Load}}^{E}$ is the electrical load, $P_{\text{chill}}^{e}$ is the electrical power consumed by the electric chiller, $P_{\text{Ch}}^{\text{EV}}$ is the electrical power consumed by the PHEV in charging its battery, $P_{\text{high.DR}}^{E}$ is the electrical load shifted by DSM to peak hours, $P_{\text{in}}^{E}$ is the electrical power purchased from the grid, $P_{\text{CHP}}^{e}$ is the electrical power produced by the CHP, $P_{\text{wind}}^{e}$ is the electrical power produced by the WT, $P_{\text{pv-final}}^{e}$ is the final electrical power produced by the PV panels, $P_{\text{low.DR}}^{E}$ is the electrical load shifted by DSM to off-peak hours, and $P_{\text{Dis}}^{\text{EV}}$ is the electrical power available from the PHEV battery during its discharge.

$${\text{Load}}^{H}(t,s)+P_{\text{ac}}^{H}(t,s)+P_{\text{high.DR}}^{H}(t,s)=P_{\text{CHP}}^{H}(t,s)+P_{\text{Boil}}^{H}(t,s)+P_{\text{low.DR}}^{H}(t,s)$$ (21)

where ${\text{Load}}^{H}$ is the heating load, $P_{\text{ac}}^{H}$ is the heating power consumed by the absorption chiller, $P_{\text{high.DR}}^{H}$ is the shifted heating power by DSM to peak hours, $P_{\text{CHP}}^{H}$ is the heating power produced by the CHP, $P_{\text{Boil}}^{H}$ is the heating power produced by the boiler, and $P_{\text{low.DR}}^{H}$ is the shifted heating power by DSM to off-peak hours.

$${\text{Load}}^{C}(t,s)+P_{\text{high.DR}}^{C}(t,s)=P_{\text{ac}}^{C}(t,s)+P_{\text{ec}}^{C}(t,s)+P_{\text{low.DR}}^{C}(t,s)$$ (22)

where ${\text{Load}}^{C}$ is the cooling load, $P_{\text{high.DR}}^{C}$ is the shifted cooling power by DSM to peak hours, $P_{\text{ac}}^{C}$ is the cooling power produced by the absorption chiller, $P_{\text{ec}}^{C}$ is the cooling power produced by the electric chiller, and $P_{\text{low.DR}}^{C}$ is the shifted cooling power by DSM to off-peak hours.

### 3.6 DSM constraints

DSM enables the shifting of controllable loads from peak to off-peak hours to lower the operational costs of the REH without compromising consumer comfort [82]. Controllable devices in the SREH allow for required shifts without compromising performance. This work computes the operational cost over a 24-hour period, and we must add the load shed from one hour to the next to maintain the same load usage over the 24-hour horizon. This paper applies DSM to electric, heating, and cooling loads. The DSM on electrical load is given by Eqs. 23 to (26).

$$\sum_{t=1}^{24}P_{\text{high.DR}}^{E}(t,s)=\sum_{t=1}^{24}P_{\text{low.DR}}^{E}(t,s)$$ (23)

$$0\leq P_{\text{high.DR}}^{E}(t,s)\leq P_{\text{DR-high}}^{E}\cdot {\text{Load}}^{E}(t,s)\cdot I_{\text{high}}^{E}(t,s)$$ (24)

$$0\leq P_{\text{low.DR}}^{E}(t,s)\leq P_{\text{DR-low}}^{E}\cdot {\text{Load}}^{E}(t,s)\cdot I_{\text{low}}^{E}(t,s)$$ (25)

$$0\leq I_{\text{high}}^{E}(t,s)+I_{\text{low}}^{E}(t,s)\leq 1$$ (26)

where $P_{\text{high.DR}}^{E}$ is the electrical load shifted to peak hours, $P_{\text{low.DR}}^{E}$ is the electrical load shifted to off-peak hours, $P_{\text{DR-high}}^{E}$ and $P_{\text{DR-low}}^{E}$ are scaling factors for the electrical load that can be shifted to peak and off-peak hours, respectively. $I_{\text{high}}^{E}$ and $I_{\text{low}}^{E}$ are binary variables determining if a load is shifted to peak hour or off-peak hour, respectively.

The DSM on heating load is given by Eqs. 27 to (30).

$$\sum_{t=1}^{24}P_{\text{high.DR}}^{H}(t,s)=\sum_{t=1}^{24}P_{\text{low.DR}}^{H}(t,s)$$ (27)

$$0\leq P_{\text{high.DR}}^{H}(t,s)\leq P_{\text{DR-high}}^{H}\cdot {\text{Load}}^{H}(t,s)\cdot I_{\text{high}}^{H}(t,s)$$ (28)

$$0\leq P_{\text{low.DR}}^{H}(t,s)\leq P_{\text{DR-low}}^{H}\cdot {\text{Load}}^{H}(t,s)\cdot I_{\text{low}}^{H}(t,s)$$ (29)

$$0\leq I_{\text{high}}^{H}(t,s)+I_{\text{low}}^{H}(t,s)\leq 1$$ (30)

where $P_{\text{high.DR}}^{H}$ is the heating load shifted to peak hours, $P_{\text{low.DR}}^{H}$ is the heating load shifted to off-peak hours, $P_{\text{DR-high}}^{H}$ and $P_{\text{DR-low}}^{H}$ are scaling factors for the heating load that can be shifted to peak and off-peak hours, respectively. $I_{\text{high}}^{H}$ and $I_{\text{low}}^{H}$ are binary variables determining if a load is shifted to peak hour or off-peak hour, respectively.

The DSM on cooling load is given by Eqs. 31 to (34).

$$\sum_{t=1}^{24}P_{\text{high.DR}}^{C}(t,s)=\sum_{t=1}^{24}P_{\text{low.DR}}^{C}(t,s)$$ (31)

$$0\leq P_{\text{high.DR}}^{C}(t,s)\leq P_{\text{DR-high}}^{C}\cdot {\text{Load}}^{C}(t,s)\cdot I_{\text{high}}^{C}(t,s)$$ (32)

$$0\leq P_{\text{low.DR}}^{C}(t,s)\leq P_{\text{DR-low}}^{C}\cdot {\text{Load}}^{C}(t,s)\cdot I_{\text{low}}^{C}(t,s)$$ (33)

$$0\leq I_{\text{high}}^{C}(t,s)+I_{\text{low}}^{C}(t,s)\leq 1$$ (34)

where $P_{\text{high.DR}}^{C}$ is the cooling load shifted to peak hours, $P_{\text{low.DR}}^{C}$ is the cooling load shifted to off-peak hours, $P_{\text{DR-high}}^{C}$ and $P_{\text{DR-low}}^{C}$ are scaling factors for the cooling load that can be shifted to peak and off-peak hours, respectively. $I_{\text{high}}^{C}$ and $I_{\text{low}}^{C}$ are binary variables determining if a load is shifted to peak hour or off-peak hour, respectively.

### 3.7 PHEV modeling

In this study, a DC/AC converter connects the PHEV’s battery to the SREH, enabling either one-way or two-way electrical power exchange, depending on the situation. The PHEV is available at home for some period of time depending upon the arrival and departure times of the consumer. The level of charge of PHEV after the arrival time depends upon the traveling distance of the consumer. The power usage of the PHEV depends on the driving distance and efficiency of the vehicle and is given by (35).

$$P_{\text{consp}}^{\text{PHEV}}=\eta_{v}\cdot DD$$ (35)

where $P_{\text{consp}}^{\text{PHEV}}$ is the electrical power consumed by the PHEV in traveling, $\eta_{v}$ is the mileage efficiency of the PHEV, and *DD* is the driving distance traveled by the PHEV.

The charging or discharging rate of PHEV must not exceed the maximum charge or discharge allowable limit of the PHEV battery and is given by Eqs. 36 and (37), respectively.

$$P_{\text{Ch}}^{\text{PHEV}}(t,s)\leq P_{\text{max}}^{\text{Ch}}$$ (36)

$$P_{\text{Dis}}^{\text{PHEV}}(t,s)\leq P_{\text{max}}^{\text{Dis}}$$ (37)

where $P_{\text{Ch}}^{\text{PHEV}}$ and $P_{\text{Dis}}^{\text{PHEV}}$ are the charging and discharging power of the PHEV battery, and $P_{\text{max}}^{\text{Ch}}$ and $P_{\text{max}}^{\text{Dis}}$ are the maximum limits of charging and discharging of the PHEV battery, respectively.

The electrical energy stored in a PHEV’s battery must not exceed the battery’s allowable limit and is given by (38).

$$0\leq P_{\text{elec}}^{\text{PHEV}}(t,s)\leq {\text{Cap}}^{\text{PHEV}}$$ (38)

where $P_{\text{elec}}^{\text{PHEV}}$ is the electrical power stored in the PHEV battery at any interval of time, and ${\text{Cap}}^{\text{PHEV}}$ is the maximum capacity of the PHEV battery.

For the comfort of the consumer, the charge level of the battery of a PHEV must be at least 50% of the maximum capacity when the consumer departs, which is given by (39).

$$P_{\text{elec}}^{\text{PHEV}}(\text{DT}(t,s)-1)=0.5\cdot {\text{Cap}}^{\text{PHEV}}$$ (39)

The schedule of charge of PHEV is calculated by (40).

$${\text{SOC}}^{\text{PHEV}}(t,s)=\frac{P_{\text{elec}}^{\text{PHEV}}(t,s)}{{\text{Cap}}^{\text{PHEV}}}$$ (40)

where ${\text{SOC}}^{\text{PHEV}}$ is the schedule of charge of the PHEV battery at any interval of time.

The SOC of the PHEV must be in the allowable range according to its technical specifications. This is given in (41).

$${\text{SOC}}_{\text{min}}^{\text{PHEV}}\leq {\text{SOC}}^{\text{PHEV}}(t,s)\leq {\text{SOC}}_{\text{max}}^{\text{PHEV}}$$ (41)

where ${\text{SOC}}_{\text{min}}^{\text{PHEV}}$ and ${\text{SOC}}_{\text{max}}^{\text{PHEV}}$ are the minimum and maximum schedule of charge of the PHEV battery, respectively.

The difference between a regular battery and a PHEV battery is that a regular battery is available all day, making it easier to model. In contrast, a PHEV battery is only available when the vehicle is at the building. Additionally, a PHEV battery discharges during travel, whereas a conventional battery only discharges to meet the demands of the REH during peak hours. There are also additional constraints associated with a PHEV battery, such as the need for it to be fully charged before departure time to ensure the comfort level of the driver. These additional constraints need to be managed effectively by an intelligent charging mechanism.

## 4 Methodology

Energy systems frequently employ MILP modeling for complicated systems. A three-step iterative technique is commonly employed to design MILPs [83]. The initial stage entails delineating a collection of decision factors that signify the options requiring optimization within the framework. Often, the second phase involves defining the model’s restrictions, and the third step requires defining the goal function. However, it can execute these two processes in any order [84].

During the modeling building process, it frequently becomes apparent that the initially specified collection of variables for decision-making is insufficient. It is often necessary to explicitly describe choice factors that seem like implicit outcomes of other choices. The incorporation of additional factors following a failed endeavor to establish limits and objectives constitutes the “loop” in the method [85]. The accurate description of decision factors may prove particularly complex when modeling using integer parameters. By using binary values in a framework, we can represent binary choices, enforce conditional assertions, and incorporate certain nonlinearities into the system, all of which we can convert into a comparable MILP.

The GAMS is a sophisticated computing framework for scientific optimization. The purpose of GAMS is to simulate and resolve optimization challenges that are linear, unpredictable, and mixed-integer. Users can construct extensive, stable simulations, adaptable to new circumstances, thanks to the system’s design for intricate and massive modeling tasks. The system is accessible on several computer systems. Models are transferable across many platforms [86]. The concept of a logical methodology for representing, manipulating, and solving extensive mathematical problems integrates traditional and contemporary concepts into a coherent and analytically feasible framework. The use of generator arrays in linear programming highlighted the importance of consistently labeling rows and columns. The link to the nascent relational data paradigm became apparent. Knowledge of conventional coding languages for managing name-spaces naturally fosters a mindset oriented toward sets and tuples, which subsequently results in the database system [87].

**Fig 6:**
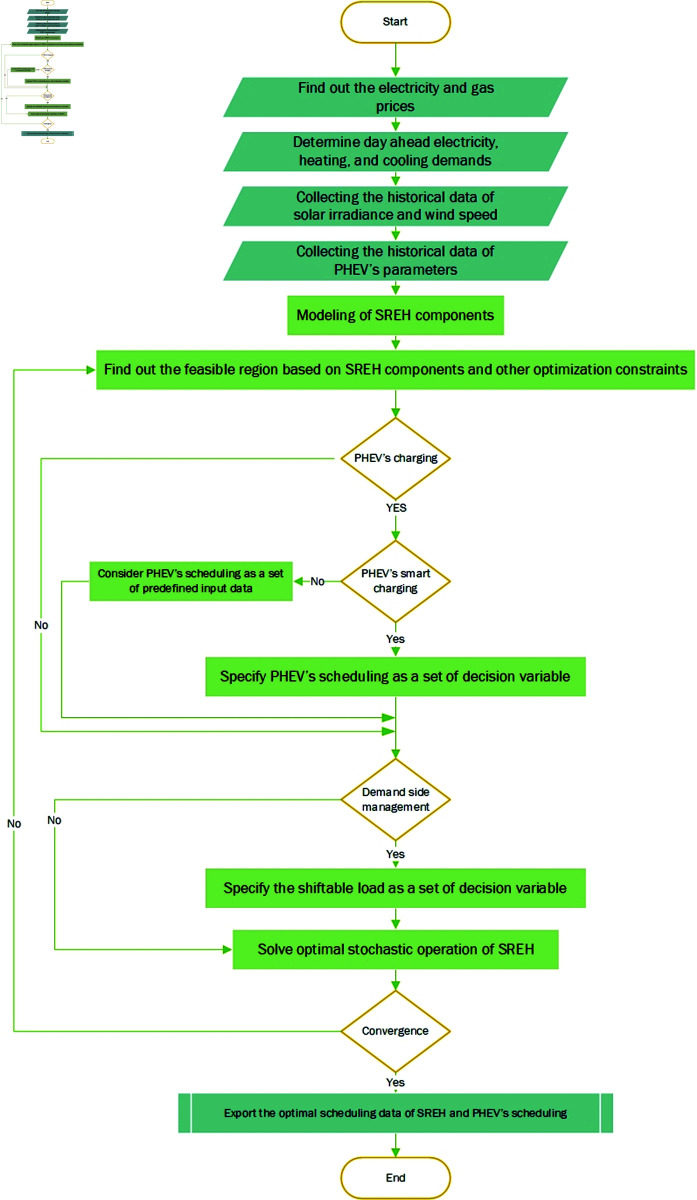
Flow chart of the proposed method for optimal operation of SREH in the presence of PHEV.

The proposed method for the optimization of SREH in the presence of PHEV with DSM is shown in Fig. 6. The optimization of REH starts with the collection of data like electricity price, gas price, electric demand, heating demand, and cooling demand. For stochastic modeling of solar and wind energy, historical data on solar irradiance and wind speed is essential. Additionally, accurate modeling of PHEV availability and charging requires historical data on parameters such as departure time, arrival time, and traveling distance. Once all the relevant data has been gathered, the different parts needed to change one type of energy into another in REH are modeled as a MILP and solved with GAMS software. Then, based upon the constraints of REH components, like maximum and minimum input or output, its feasible operating region is identified. Next, we incorporate PHEV’s charging, PHEV’s smart charging, and DSM modules to enhance REH’s intelligence. Finally, this work solved the entire SREH model, export the results, and conduct further investigations.

## 5 Simulation and case studies

To check the impact of the integration of PHEVs into the REH and the significance of smart charging in the SREH environment, four different cases are discussed in this section.

1. **Case Study-I:** The first case study is the foundational scenario excluding the participation of PHEV in REH.
2. **Case Study-II:** The second case study involves PHEV in REH.
3. **Case Study-III:** The third scenario pertains to the intelligent charging of PHEVs in smart residential energy network.
4. **Case Study-IV:** The fourth case study examines the smart PHEVs within the smart residential energy network in conjunction with DSM.

### 5.1 Simulation setup

**Fig 7:**
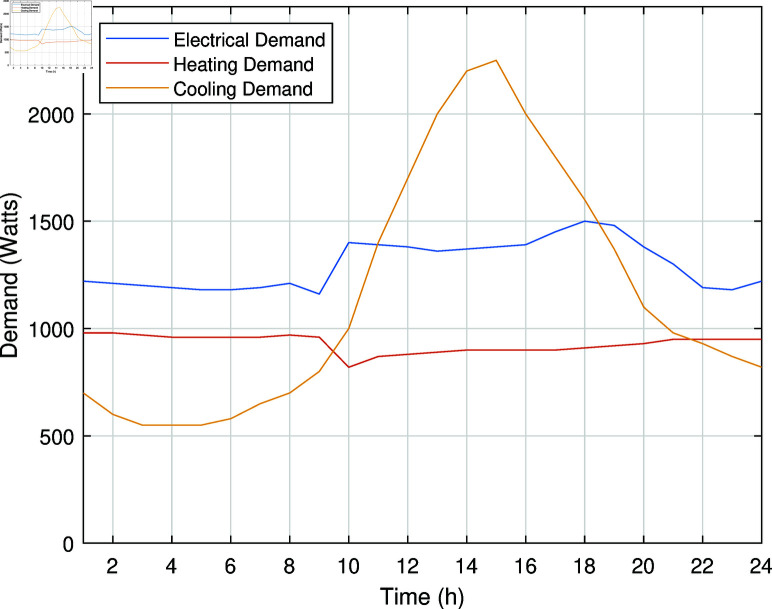
Electrical, heating, and cooling demands for SREH.

The proposed SREH model for scenario-based optimal scheduling to a residential building that includes two RESs (solar and wind), a CCHP unit, an electric chiller, a boiler, a PHEV, and various electrical, heating, and cooling loads. This study considers 10% of the load to be shiftable for DSM. The electrical, heating, and cooling demands considered for SREH are illustrated in Fig. 7.

In this work, the PHEV considered for simulation purposes is the Chevrolet Volt, which has a 33000 Wh electric battery. The detailed technical features of this PHEV are given in Table 2.

**Table 2:** Technical features of PHEV battery.

Parameter	Unit	Value
Name of PHEV	Chevrolet	Volt
Battery Capacity	kWh	33
Max. charging/discharging rate	kW	3.3
Plug out time	h	07
Plug in time	h	19
Consumption per km	kWh	0.1
Battery lower limit	%	20
Battery upper limit	%	100

For the REH simulation, Table 3 shows the features of the EH central devices, Table 4 shows the features of the PV modules, and Table 5 shows the features of the wind turbines [76].

**Table 3:** Technical features of central devices.

Parameter	Unit	Value
Efficiency of gas turbines in heat generation	%	40
Efficiency of gas turbines in electricity generation	%	35
Boiler efficiency	%	80
Absorption chiller COP	%	130
Electric Chiller efficiency	%	80
Maximum heat power generated from boiler	kW	1.8
Transformer efficiency	%	95
Maximum power generated from CHP	kW	1.2

**Table 4:** Technical features of PV module.

Parameter	Unit	Value
Maximum PV module output power	kW	0.4
Derating factor of PV module	%	80
Solar irradiance at standard test conditions	${\text{kW/m}}^{2}$	1
Coefficient of temperature	%/°C	-0.5
Temperature of PV cell	°C	60
Temperature of PV cell under STC	°C	25

**Table 5:** Technical features of wind turbine.

Parameter	Unit	Value
Maximum WT output	kW	0.4
Cut in speed of WT	m/s	4
Cut out speed of WT	m/s	22
Rated speed of WT	m/s	10

**Fig 8:**
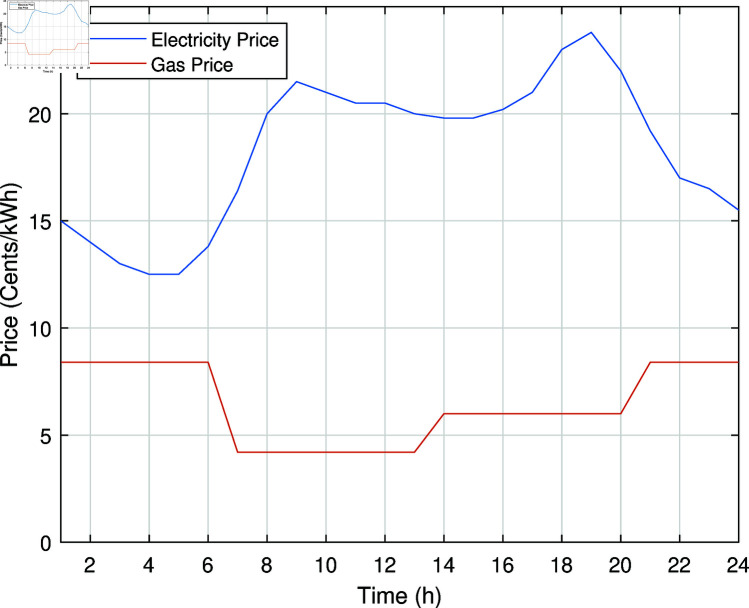
Electricity and gas prices.

The electricity and natural gas price used for simulation is taken from [88] and are shown in Fig. 8.

## 6 Results

The present work formulate the overall problem as MILP and solve it in the GAMS environment, incorporating RES. It simulate four different scenarios to evaluate the SREH’s performance in the context of PHEV.

### 6.1 Case Study-I

In case study I, this work investigate the optimal operation of REH, which includes RES (both solar and wind energy), CHP, EC, AC, a boiler, and three types of loads. The electricity and gas procured from the grid to fulfill demand are shown in Table 6 (Case Study-I(a)). Given the lower price of gas compared to electricity, we purchase more gas than electricity to ensure optimal operation of the REH. The REH requires more gas from 11:00 to 19:00, but it restricts its procurement to 2.4 kW to prevent exceeding the upper limit. During this period, the dependency on the electricity increases. The total operating cost of REH in Case Study-I is 7675.00 USD for a 24-hour period.

**Table 6:** Results for Case Study-I.

Case Study-I (a)												
Hour	1	2	3	4	5	6	7	8	9	10	11	12
Electricity Purchased (Watts)	652.6	600	568.4	557.9	369.4	318.7	451.2	646	719	867.5	968.7	1182.5
Gas Purchased (Watts)	1882.2	1882.2	1811.1	1800	1800	1800	1800	1811.1	1800	1644.4	2400	2400
Hour	13	14	15	16	17	18	19	20	21	22	23	24
Electricity Purchased (Watts)	1591.9	1888	1991.1	1708.2	1503.7	1403.6	1234.7	1157.8	910.8	717.8	682.1	703.1
Gas Purchased (Watts)	2400	2400	2400	2400	2400	2400	2400	2084.1	1788.8	1788.8	1788.8	1788.8
**Case Study-I (b)**												
Hour	1	2	3	4	5	6	7	8	9	10	11	12
Electricity from CHP (Watt)	480	480	480	480	480	480	480	480	480	480	480	480
Electricity from grid (Watt)	589	541.5	513	503.4	333.4	287.7	407.2	583	648.8	782.9	874.3	1067.2
RES power production (Watt)	400	400	400	400	569	629.1	541.3	396.2	316.9	495.8	614	780.3
Electric chiller consumption (Watt)	0	0	0	0	0	0	0	0	0	179.5	483	786.5
Heat pump consumption (Watt)	280	240	220	220	220	232	260	280	320	400	400	400
Hour	13	14	15	16	17	18	19	20	21	22	23	24
Electricity from CHP (Watt)	480	480	480	480	480	480	480	480	480	480	480	480
Electricity from grid (Watt)	1436.7	1703.9	1796.9	1541.6	1357.1	1266.7	1114.3	1045	822	647.8	615.6	634.6
RES power production (Watt)	750.7	733.8	708.5	674.6	678.9	578.4	435.7	200	346.7	400	400	400
Electric chiller consumption (Watt)	990	1040	790	590	393.5	167	0	0	0	0	0	0
Heat pump consumption (Watt)	400	400	400	400	400	400	400	400	392	372	348	328
**Case Study-I (c)**												
Hour	1	2	3	4	5	6	7	8	9	10	11	12
Gas turbine heating power (Watt)	420	420	420	420	420	420	420	420	420	420	420	420
Boiler heating power (Watt)	560	560	550	540	540	540	540	550	540	400	450	460
Heat pump heating power (Watt)	0	0	0	0	0	0	0	0	0	0	0	0
Hour	13	14	15	16	17	18	19	20	21	22	23	24
Gas turbine heating power (Watt)	420	420	420	420	420	420	420	420	420	420	420	420
Boiler heating power (Watt)	470	480	480	480	480	490	500	510	530	530	530	530
Heat pump heating power (Watt)	0	0	0	0	0	0	0	0	0	0	0	0
**Case Study-I (d)**												
Hour	1	2	3	4	5	6	7	8	9	10	11	12
Absorption chiller cooling power (Watt)	0	0	0	0	0	0	0	0	0	0	220.5	217
Heat pump cooling power (Watt)	700	600	550	550	550	580	650	700	800	1000	1000	1000
Electric chiller cooling power (Watt)	0	0	0	0	0	0	0	0	0	0	179.5	483
Hour	13	14	^′^15	16	17	18	19	20	21	22	23	24
Absorption chiller cooling power (Watt)	213.5	210	210	210	210	206.5	203	100	0	0	0	0
Heat pump cooling power (Watt)	1000	1000	1000	1000	1000	1000	1000	1000	980	930	870	820
Electric chiller cooling power (Watt)	483	786.5	990	1040	790	590	393.5	167	0	0	0	0

The electrical demand of REH and electrical energy consumed by electric chiller and EHP is fulfilled by the electrical energy purchased from the grid, the electrical energy produced from RES (solar and wind), and the electrical energy generated by the CHP, as shown in Table 6 (Case Study-I(b)). The CHP utilizes the lower cost of gas compared to electricity, operates at its peak efficiency, and generates 480 watts of electrical power from the gas. The electrical power generated from RES is non-dispatchable and available only when sunlight or wind is available.

The heating demand of REH is met by gas turbine and boiler only, as shown in Table 6 (Case Study-I(c)). Depending upon the weather and load condition, EHP can produce heating power by changing its mode of operation, but in this case, it produces only cooling energy because cooling demand is significantly higher than the heating demand. EHP cannot produce heating and cooling power simultaneously.

The cooling demand of the REH is fulfilled by an absorption chiller, an EHP, and an electric chiller, as illustrated in Table 6 (Case Study-I(d)). The EHP generates cooling power continuously due to its low cost, while electric chillers only activate during peak hours to meet the cooling demand, as they require expensive electricity.

### 6.2 Case Study-II

This case study incorporates the PHEV into the REH, complementing the components already present in case 1. We assume that PHEVs depart from their residence at 7:00 and reach their destination at 17:00, using half of their battery capacity during the journey. In this scenario, the unmanaged charging of PHEV leads to an increase in electrical demand during peak hours. This situation results in an increase in the operating cost of REH compared to the first case, where PHEV was not present. To meet the additional electrical demand due to PHEV charging, the amount of purchased electricity and gas increases, as shown in Table 7 (Case Study-II(a)).

**Table 7:** Results for Case Study-II.

Case Study-II (a)												
Hour	1	2	3	4	5	6	7	8	9	10	11	12
Electricity Purchased (Watts)	652.6	600	568.4	557.9	369.4	318.7	556.4	551.2	603.2	730.7	842.4	956.2
Gas Purchased (Watts)	1882.2	1882.2	1811.1	1800	1800	1800	1800	1811.1	1800	1644.4	2400	2400
Hour	13	14	15	16	17	18	19	20	21	22	23	24
Electricity Purchased (Watts)	1581.4	1898.5	1999.4	1602.9	1503.7	1403.6	1234.7	1157.8	216.1	23.15	0	273.6
Gas Purchased (Watts)	2400	2400	2400	2400	2400	2400	2400	2084.1	1788.8	1788.8	1788.8	1788.8
**Case Study-II (b)**												
Hour	1	2	3	4	5	6	7	8	9	10	11	12
Electricity from CHP (Watt)	480	480	480	480	480	480	480	480	480	480	480	480
Electricity from grid (Watt)	620.0	570	540	529.9	350.9	302.8	528.6	523.7	573	694.1	800.3	908.4
RES power production (Watt)	400	400	400	400	569	629.1	541.3	396.2	316.9	495.8	614	780.3
Electric chiller consumption (Watt)	0	0	0	0	0	0	0	0	0	224.3	603.7	983.1
Heat pump consumption (Watt)	280	240	220	220	220	232	260	280	320	400	400	400
Hour	13	14	15	16	17	18	19	20	21	22	23	24
Electricity from CHP (Watt)	480	480	480	480	480	480	480	480	480	480	480	480
Electricity from grid (Watt)	1502.3	1803.6	1899.5	1522.8	1428.5	1333.4	1173.0	1100	205.3	22	0	259.9
RES power production (Watt)	750.7	733.8	708.5	674.6	678.9	578.4	435.7	200	346.7	400	400	400
Electric chiller consumption (Watt)	1273.5	1300	987.5	737.5	491.8	208.7	0	0	0	0	0	0
Heat pump consumption (Watt)	400	400	400	400	400	400	400	400	392	372	348	328
**Case Study-II (c)**												
Hour	1	2	3	4	5	6	7	8	9	10	11	12
Gas turbine heating power (Watt)	360	360	360	360	360	360	4200	420	420	420	420	420
Boiler heating power (Watt)	620	620	610	600	600	600	540	550	540	400	40	460
Heat pump heating power (Watt)	0	0	0	0	0	0	0	0	0	0	0	0
Hour	13	14	15	16	17	18	19	20	21	22	23	24
Gas turbine heating power (Watt)	420	420	400	400	400	400	400	420	360	360	360	360
Boiler heating power (Watt)	470	500	500	500	500	510	520	510	590	590	590	590
Heat pump heating power (Watt)	0	0	0	0	0	0	0	0	0	0	0	0
**Case Study-II (d)**												
Hour	1	2	3	4	5	6	7	8	9	10	11	12
Absorption chiller cooling power (Watt)	0	0	0	0	0	0	0	0	0	0	220.5	217
Heat pump cooling power (Watt)	700	600	550	550	550	580	650	700	800	1000	1000	1000
Electric chiller cooling power (Watt)	0	0	0	0	0	0	0	0	0	0	179.5	483
Hour	13	14	^′^15	16	17	18	19	20	21	22	23	24
Absorption chiller cooling power (Watt)	213.5	210	210	210	210	206.5	203	100	0	0	0	0
Heat pump cooling power (Watt)	1000	1000	1000	1000	1000	1000	1000	1000	980	930	870	820
Electric chiller cooling power (Watt)	483	786.5	990	1040	790	590	393.5	167	0	0	0	0

The electric, heating, and cooling portions of REH in Case Study-II are shown in Table 7 (Case Study-II(b,c, and d)), respectively. The unmanaged charging of PHEVs creates an increased burden on REH, mainly in peak hours. The REH’s operational cost rises to 7723.70 USD due to the ineffective use of PHEV’s stored electrical energy during peak hours. Therefore, adding a PHEV to the REH without any management increases the operational cost by 0.63%.

**Fig 9:**
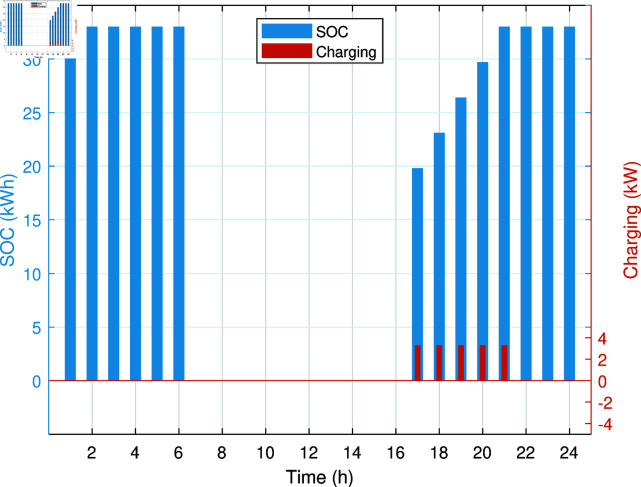
SOC of PHEV battery and its charging in Case Study-II.

The SOC and charging of PHEV without any management are shown in Fig. 9. The PHEV gets charged immediately after coming home, irrespective of the fact that it is peak hour time. The unmanaged charging of PHEV has caused a significant increase in the energy costs due to higher dependency on costly electrical energy and gas in peak hours. In addition, REH experiences increased electrical peak demands because charging of the PHEV is not coordinated. The ability of PHEV to act as a storage resource is untapped in this case, thus reducing the operational efficiency of REH. Thus, adding a PHEV to the REH without any smart management system has increased operational cost, increased peak demands, and decreased reliability. To improve this situation, implementing a smart charging strategy could significantly reduce the operational cost of REH and also improve the flow of energy within REH, which is discussed in the next case study.

### 6.3 Case Study-III

**Fig 10:**
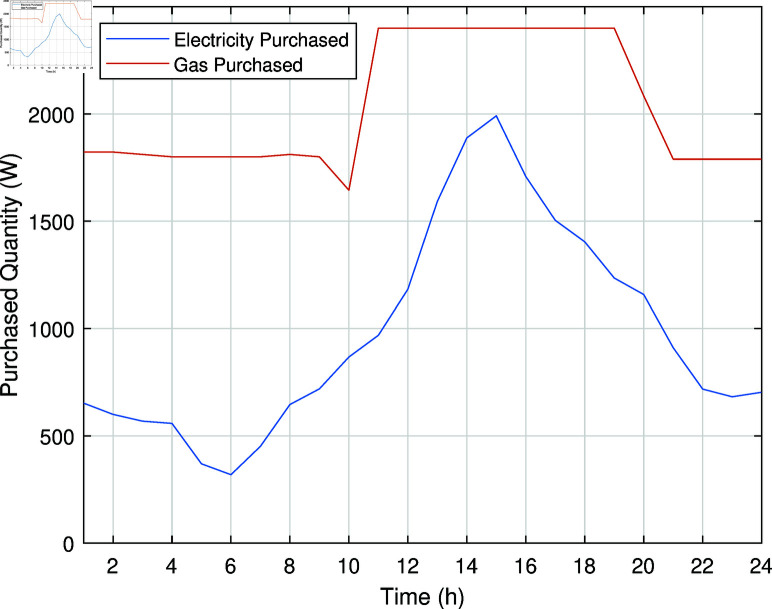
Electricity and natural gas purchased from the grid in Case Study-III.

In Case Study-III, a smart management system is implemented for the charging of PHEV, which not only manages peak/off peak hours but also provides electrical energy to the REH by discharging when necessary. This approach optimizes the charging and discharging of PHEV battery to minimize the operational costs of REH. The smart charging/discharging system ensures that electrical energy demand is distributed more evenly throughout the day, reducing peaks and leveraging off-peak charging. It is assumed that PHEV leaves home at 7:00 with a full charge and arrives home after traveling a distance at 17:00 with 50% charge left. The electricity and gas purchased from the grids in Case Study-III are illustrated in Fig. 10.

**Fig 11:**
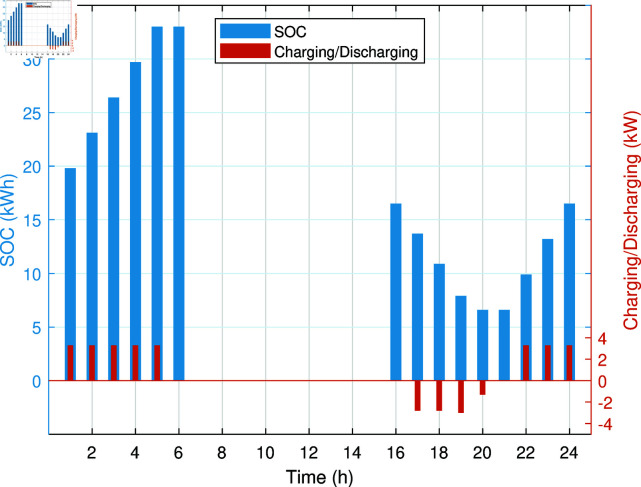
SOC and charging/discharging of PHEV battery in Case Study-III.

**Fig 12:**
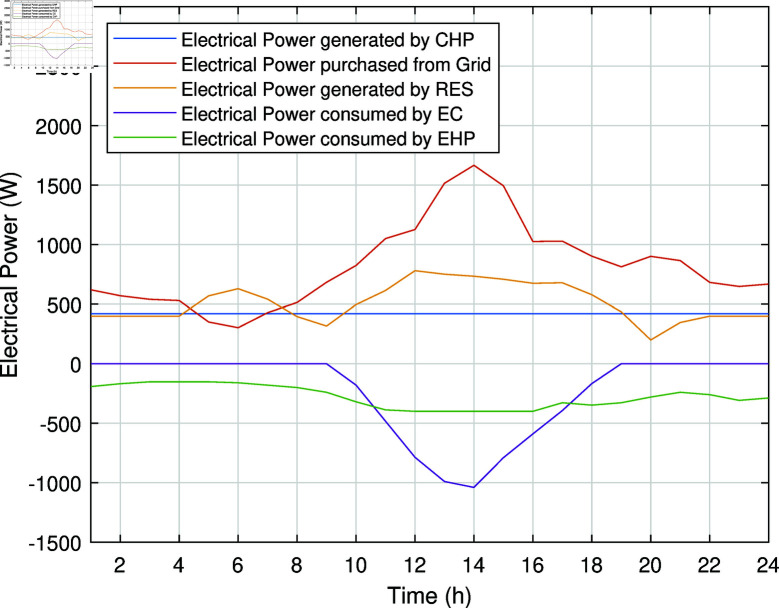
Electrical power of SREH in Case Study-III.

**Fig 13:**
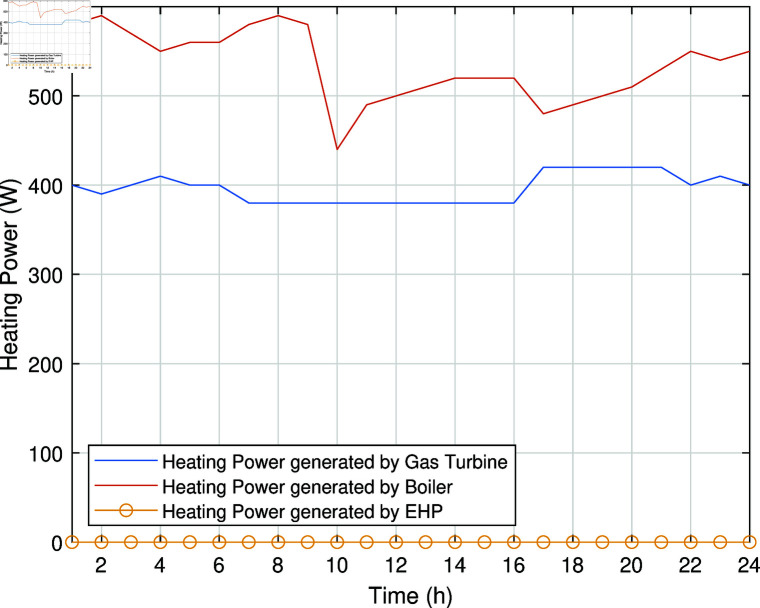
Heating power of SREH in Case Study-III.

**Fig 14:**
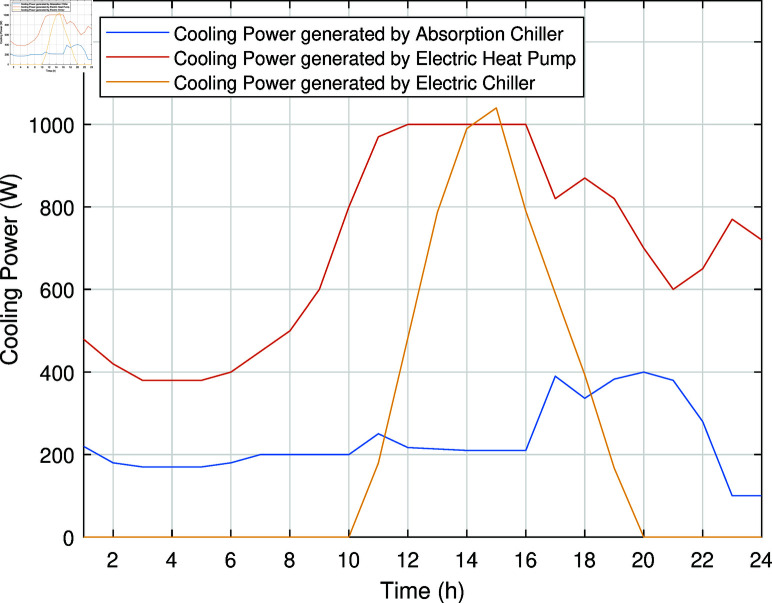
Cooling power of SREH in Case Study-III.

The smart charging of PHEV makes it possible to discharge the battery of PHEV in peak hours and charge it back in off-peak hours, as shown in Fig. 11. Instead of charging immediately upon arrival, as in the previous case, the PHEV battery is discharged during peak hours to supply energy to REH during high tariffs and demand. By extracting a portion of electrical energy from the battery during peak hours and charging it during off-peak hours, we significantly reduce the operational cost of SREH. The battery is charged again during off-peak hours when electricity is cheap, ensuring that it is ready for use again the next day. Charging the PHEV during off-peak hours allows the REH to take advantage of lower energy rates. The PHEV battery functions as both an energy storage system and a power source, increasing the flexibility and efficiency of the REH. In case Study-III, smart charging reduces the operational cost of SREH to 7523.10 USD from 7723.70 USD, indicating a reduction of approximately 2.59%. The electric, heating and cooling portion of REH in Case Study III is shown in Figs 12-14. This case highlights the importance of integrating smart energy management systems for PHEVs in achieving a cost-effective and efficient REH.

### 6.4 Case Study-IV

**Fig 15:**
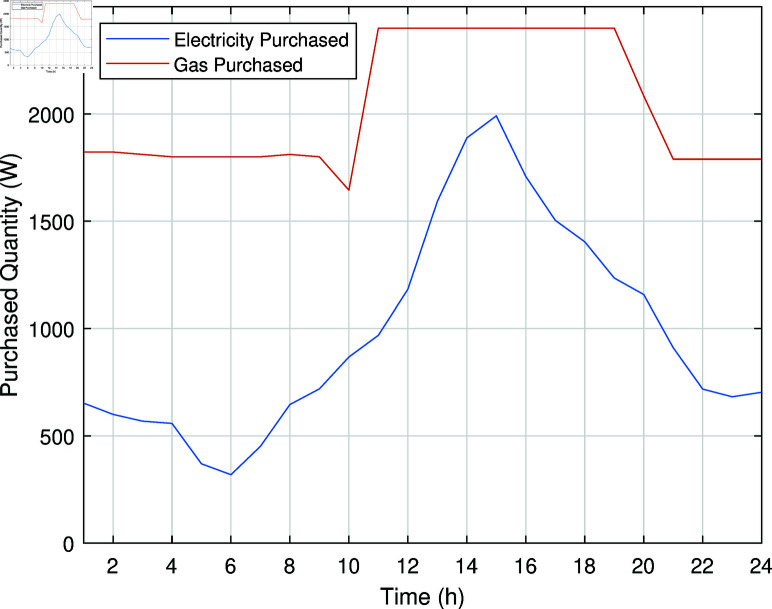
Electricity and natural gas purchased from the grid in Case Study-IV.

**Fig 16:**
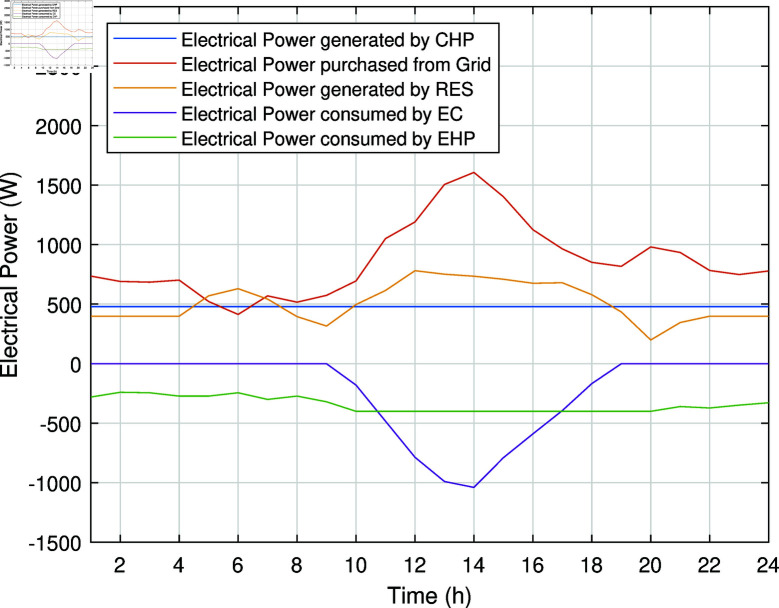
Electrical power of SREH in Case Study-IV.

**Fig 17:**
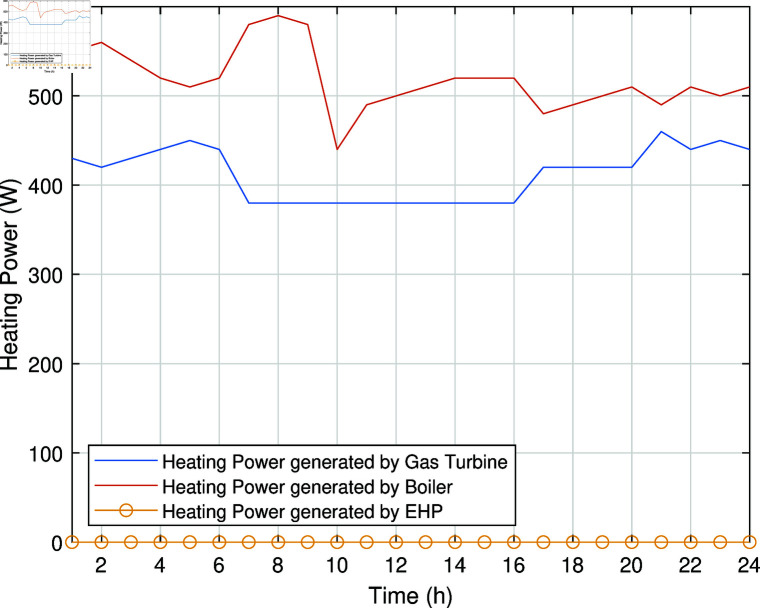
Heating power of SREH in Case Study-IV.

**Fig 18:**
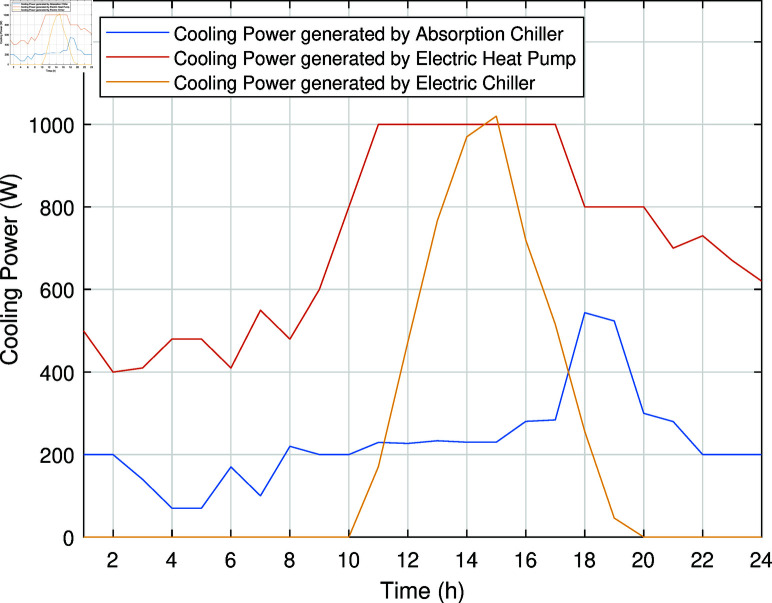
Cooling power of SREH in Case Study-IV.

**Fig 19:**
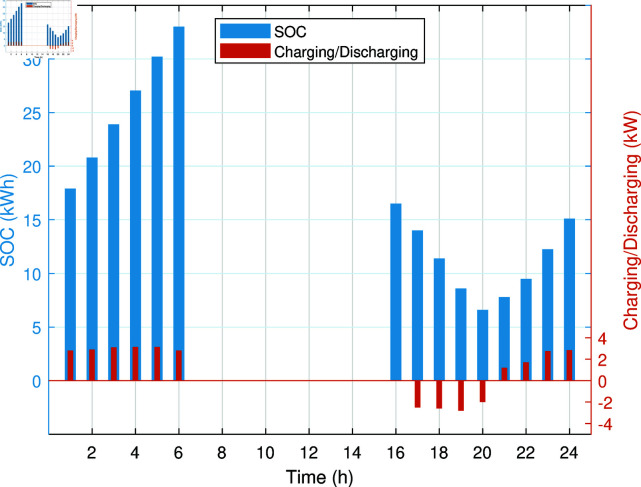
SOC and charging/discharging of PHEV battery in Case Study-IV.

In Case Study IV, the DSM technique is introduced to further optimize the operation of the REH. DSM involves actively managing the energy demand by shifting some loads from peak hours to off-peak hours, reducing the dependance on grid energy during high-cost periods. We assume that 10% of the loads are shiftable. These shiftable loads include non-essential or flexible energy-consuming appliances and systems that do not need to operate during peak hours. The DSM strategy redistributes a portion of the energy demand by delaying or advancing operations to occur during off-peak hours, when electricity and gas prices are lower. This reduces the dependency on purchased energy during peak hours when tariffs are higher. The ability to shift portions of the load from peak to off-peak hours decreases dependence on purchased electricity and gas during peak times, leading to further cost reductions. Fig. 15 illustrates the electricity and gas purchased from the respective grids in Case Study IV. This load shifting by DSM results in a further reduction of the operating costs of the REH to 7439.68 USD, reflecting an additional decrease of approximately 1.11%. The electric, heating, and cooling portion of REH in Case Study-IV is shown in Figs 16 to 18. The total operational cost reduction from Case Study II to Case Study IV is significant, highlighting the cumulative benefits of smart energy management techniques. Shifting some portions of the load also enhances the flexibility in charging and discharging of the PHEV battery, as demonstrated in Fig. 19. The application of DSM ensures that load is spread more evenly across the day, reducing peaks and taking advantage of off-peak energy rates. This case highlights the importance of DSM as a key strategy for reducing energy costs and improving the efficiency of renewable energy systems in REH.

### 6.5 Cases summary

The first case is a base case in which the total operational cost of REH is 7675.00 USD. The second case incorporates the PHEV without any smart charging mechanism, which increases the operational cost of REH by 0.63% due to the additional charging of PHEV. The third case involves the smart charging mechanism, which takes care of peak/off-peak hours and smartly charges/discharges the PHEV. The employed smart charging mechanism decreases the cost of SREH by 1.98% while also charging the PHEV and increases the reliability of REH as well. The fourth case involves DSM in addition to smart charging, which results in a further decrease in cost by 3.06%. A summary of these results is shown in Table 8.

**Table 8:** Operational cost analysis of SREH under different case studies.

Case Study	PHEV Inclusion	Smart Charging of PHEV	Demand Side Management	Total Cost (USD)	% Change
I	-	-	-	7675.00	-
II	✓	-	-	7723.70	+0.63
III	✓	✓	-	7523.10	-1.98
IV	✓	✓	✓	7439.68	-3.06

### 6.6 Discussion

The first case is the base case, containing all the basic components of the REH, such as RES, CHP, AC, EC, a boiler, and electrical, heating, and cooling loads, but without the involvement of a PHEV. After a proper sensitivity analysis of key REH parameters, the sizes of different components are carefully selected, keeping practical systems in mind. The parameters of RES and central devices are optimally selected so that they are neither too large, which would result in high investment or operational costs, nor too small, making their impact negligible. After selecting the desired configuration, the REH is optimized for its operational cost over 24 hours. The second case involves incorporating a PHEV into the REH without any smart charging mechanism to analyze the impact of adding a PHEV to the REH. The unmanaged charging of the PHEV increases the operational cost of the REH, highlighting the need for proper smart charging of the PHEV. In the third case, a smart charging mechanism is introduced that optimally charges/discharges the PHEV, taking into account the pricing of purchased electricity at any given time. The PHEV charging/discharging process is specified as a set of decision variables based on the pricing mechanism. This enables the PHEV to be charged when electricity prices are low and discharged when they are high. The flexibility of discharging the PHEV to obtain electrical energy when the system is under stress increases the stability and reliability of the REH, making it more robust and resilient. The fourth case involves DSM, which shifts some portion of the load when required. When there is increased load stress on the REH during peak hours, DSM makes it possible to optimally shift some shiftable loads to off-peak hours, reducing the need to purchase high-cost energy. This strategy not only increases the stability of the REH but also decreases its operational cost.

## 7 Conclusions

This article presents a stochastic MILP model of REH that integrates with RES in the presence of PHEV. Four case studies were conducted to assess the effectiveness of the proposed scheme. Firstly, the proposed scheme optimizes REH in the presence of RES, meeting its electrical demand through a combination of CHP, RES, and grid-purchased electricity. The results show that when the price of gas is less than the electricity price, the CHP unit generates more electrical energy from the gas to avoid the costly purchase of electricity. EHP converts the electrical energy into cooling power instead of heating energy due to the high demand for cooling energy in summer weather. Without any charging management, adding the PHEV to the REH setup causes the operating cost of REH to increase, rather than decrease, as it charges during peak hours. However, smart charging and discharging of the PHEV allow SREH to significantly reduce its operational expenses. Smart management of a PHEV battery enables the consumption of stored electrical energy during peak hours, eliminating the need to purchase it from the grid and recharging it during off-peak hours for the next day’s trip. Finally, the DSM technique is applied to all types of loads to further reduce operational costs without compromising customer comfort. In terms of practical applications, the proposed model can be implemented in smart buildings, residential apartments, and societies to optimize energy efficiency and reduce reliance on expensive grid electricity. By implementing smart energy management strategies, real-world applications of this model can significantly reduce energy costs and carbon footprints for residential consumers. To make the overall energy infrastructure more resilient and cost-effective, urban planners and policymakers can apply these insights to build sustainable microgrids that seamlessly integrate RES, PHEVs, and ESS. This study has not considered the actual degradation of batteries in PHEVs and their long-term economic implications. Future research should aim to incorporate the impact of battery degradation and its replacement costs. Investigating the integration of emerging technologies, such as hydrogen storage and vehicle-to-grid interactions, could further improve the flexibility and sustainability of the REH.

## References

[1] Ahmed I, Maaruf M, Ali A, Al-Muhaini M, Khalid M. A review on challenges and future of green charging networks for zero emission electric transportation. Energy Convers Manage X. 2025;25:100867. doi: 10.1016/j.ecmx.2024.100867

[2] Khalid M. Passivity-based nonlinear control approach for efficient energy management in fuel cell hybrid electric vehicles. IEEE Access. 2024;12:84169–84188. doi: 10.1109/ACCESS.2024.3412888

[3] Rokonuzzaman Md, Rahman S, Hannan MA, Mishu MK, Tan W-S, Rahman KS, et al. Levenberg-Marquardt algorithm-based solar PV energy integrated internet of home energy management system. Appl Energy. 2025;378:124407. doi: 10.1016/j.apenergy.2024.124407

[4] Um-e-Habiba, Ahmed I, Alqahtani M, Asif M, Khalid M. The role of energy management technologies for cyber resilient smart homes in sustainable urban development. Energy Strat Rev. 2024;56:101602. doi: 10.1016/j.esr.2024.101602

[5] Ahmed I, Asif M, Alhelou HH, Khalid M. A review on enhancing energy efficiency and adaptability through system integration for smart buildings. J Build Eng. 2024;89:109354. doi: 10.1016/j.jobe.2024.109354

[6] Ascione F, Nižetić S, Wang F. Future technologies for building sector to accelerate energy transition. Energy Build. 2025;326:115044. doi: 10.1016/j.enbuild.2024.115044

[7] Rong Y, Xu Z, Liu J, Liu H, Ding J, Liu X, et al. Du-Bus: a realtime bus waiting time estimation system based on multi-source data. IEEE Trans Intell Transport Syst. 2022;23(12):24524–39. doi: 10.1109/tits.2022.3210170

[8] Zhang J, Li H, Kong X, Zhou J, Shi G, Zang J, et al. A novel multiple-medium-ac-port power electronic transformer. IEEE Trans Ind Electron. 2023;71(7):6568–78.

[9] Ahmed I, Rehan M, Basit A, Tufail M, Ullah N, Piecha M, et al. A novel distributed approach for event-triggered economic dispatch of energy hubs under ramp-rate limits integrated with sustainable energy networks. Energy Rep. 2023;10:4097–111. doi: 10.1016/j.egyr.2023.10.078

[10] Ren K, Liu J, Wu Z, Liu X, Nie Y, Xu H. A data-driven DRL-based home energy management system optimization framework considering uncertain household parameters. Appl Energy. 2024;355:122258. doi: 10.1016/j.apenergy.2023.122258

[11] Aziz S, Ahmed I, Khan K, Khalid M. Emerging trends and approaches for designing net-zero low-carbon integrated energy networks: a review of current practices. Arab J Sci Eng. 2023;49(5):6163–85. doi: 10.1007/s13369-023-08336-0

[12] Ahmed I, Rehan M, Basit A, Hong K-S. Greenhouse gases emission reduction for electric power generation sector by efficient dispatching of thermal plants integrated with renewable systems. Sci Rep. 2022;12(1):12380. doi: 10.1038/s41598-022-15983-0 35858895 PMC9300730

[13] Khalid M, Ahmed I, AlMuhaini M, Savkin AV. A novel computational paradigm for scheduling of hybrid energy networks considering renewable uncertainty limitations. Energy Rep. 2024;11:1959–78.

[14] Syed M, Siddiqui O, Kazerani M, Khalid M. Analysis and modeling of direct ammonia fuel cells for solar and wind power leveling in smart grid applications. IEEE Access. 2024;12:46512–23. doi: 10.1109/ACCESS.2024.3376513

[15] Ahmed I, Basit A, Rehan M, Amjad A, Maaruf M, Khalid M. A resilient consensus-based energy 5.0 framework for micro-grids under ramp-rate constraints and stochastic FDI attacks. In: 2024 IEEE International Conference on Industrial Technology (ICIT). IEEE.; 2024, pp. 1–6.

[16] Salmanpour F, Yousefi H, Ehsan M. A scenario-based modelling for the long-term energy planning based on efficient energy use, economic and environmental emission reduction on national scale: a case study Iran. Energy Convers Manag. 2024;100837.

[17] Ahmed I, Rehan M, Basit A, Tufail M, Hong KS. Neuro-fuzzy and networks-based data driven model for multi-charging scenarios of plug-in-electric vehicles. IEEE Access. 2023;11:87150–87165. doi: 10.1109/ACCESS.2023.3303963

[18] Zhang H, Yu C, Zeng M, Ye T, Yue D, Dou C, et al. Homomorphic encryption based resilient distributed energy management under cyber-attack of micro-grid with event-triggered mechanism. IEEE Trans Smart Grid. 2024;15(5):5115-26.

[19] Feng J, Yao Y, Liu Z. Developing an optimal building strategy for electric vehicle charging stations: automaker role. Environ Dev Sustain. 2024:1–61.

[20] Zhang H, Yue D, Dou C, Hancke GP. PBI based multi-objective optimization via deep reinforcement elite learning strategy for micro-grid dispatch with frequency dynamics. IEEE Trans Power Syst. 2023;38(1):488–98. doi: 10.1109/tpwrs.2022.3155750

[21] Li N, Cao Y, Liu X, Zhang Y, Wang R, Jiang L, et al. An improved modulation strategy for single-phase three-level neutral-point-clamped converter in critical conduction mode. J Mod Power Syst Clean Energy. 2024;12(3):981–90. doi: 10.35833/mpce.2023.000210

[22] ALAhmad A, Verayiah R, Shareef H, Ramasamy A, Baswaimi S. Optimizing renewable energy and green technologies in distribution systems through stochastic planning of distributed energy resources. Energy Convers Manage X. 2024:100834.

[23] Ahmed I, Rehan M, Basit A, Tufail M, Hong K-S. A dynamic optimal scheduling strategy for multi-charging scenarios of plug-in-electric vehicles over a smart grid. IEEE Access. 2023;11:28992–9008. doi: 10.1109/access.2023.3258859

[24] Yi X, Lu T, Li Y, Ai Q, Hao R. Collaborative planning of multi-energy systems integrating complete hydrogen energy chain. Renew Sustain Energy Rev. 2025;210:115147. doi: 10.1016/j.rser.2024.115147

[25] Huang Z, Zhou Y, Lin Y, Zhao Y. Resilience evaluation and enhancing for China’s electric vehicle supply chain in the presence of attacks: a complex network analysis approach. Comput Ind Eng. 2024;195:110416. doi: 10.1016/j.cie.2024.110416

[26] Samadi M, Nikkhah MH, Lotfi H. Electric vehicles and the operation of multi-carrier energy systems in the smart grid: a review study. Int J Ambient Energy. 2024;45(1). doi: 10.1080/01430750.2024.2331231

[27] Zhang J, Feng X, Zhou J, Zang J, Wang J, Shi G, et al. Series–shunt multiport soft normally open points. IEEE Trans Ind Electron. 2023;70(11):10811–21. doi: 10.1109/tie.2022.3229375

[28] Yang C, Zhao Y, Li X, Zhou X. Electric vehicles, load response, and renewable energy synergy: a new stochastic model for innovation strategies in green energy systems. Renew Energy. 2025;238:121890. doi: 10.1016/j.renene.2024.121890

[29] Gao S, Chen Y, Song Y, Yu Z, Wang Y. An efficient half-bridge MMC model for EMTP-type simulation based on hybrid numerical integration. IEEE Trans Power Syst. 2024;39(1):1162–77. doi: 10.1109/tpwrs.2023.3262584

[30] El-Afifi MI, Sedhom BE, Eladl AA, Elgamal M, Siano P. Demand side management strategy for smart building using multi-objective hybrid optimization technique. Results Eng. 2024;22:102265. doi: 10.1016/j.rineng.2024.102265

[31] Lyu C, Zhan S, Zhang Y, Song Z. Synergistic two-stage optimization for multi-objective energy management strategy of integrated photovoltaic-storage charging stations. J Energy Storage. 2024;89:111665. doi: 10.1016/j.est.2024.111665

[32] Kanakadhurga D, Prabaharan N. Smart home energy management using demand response with uncertainty analysis of electric vehicle in the presence of renewable energy sources. Appl Energy. 2024;364:123062. doi: 10.1016/j.apenergy.2024.123062

[33] Jha BK, Tiwari A, Kuhada RB, Pindoriya NM. IoT-enabled smart energy management device for optimal scheduling of distributed energy resources. Electric Power Syst Res. 2024;229:110121. doi: 10.1016/j.epsr.2024.110121

[34] Du G, Li S, Cao S, Wang G, Duan J. Weekly economic scheduling of virtual power plant with electric vehicles: Deep-learning-based prediction and daily operation mode classification. Electric Power Syst Res. 2025;241:111362. doi: 10.1016/j.epsr.2024.111362

[35] Ahmed I, Rehan M, Basit A, Malik SH, Alvi U-E-H, Hong K-S. Multi-area economic emission dispatch for large-scale multi-fueled power plants contemplating inter-connected grid tie-lines power flow limitations. Energy. 2022;261:125178. doi: 10.1016/j.energy.2022.125178

[36] Zhao Z, Xu J, Lei Y, Liu C, Shi X, Lai LL. Robust dynamic dispatch strategy for multi-uncertainties integrated energy microgrids based on enhanced hierarchical model predictive control. Appl Energy. 2025;381:125141. doi: 10.1016/j.apenergy.2024.125141

[37] Eladeb A, Basem A, Sharma A, Dhawan A, Sharma P, Bouzidi M, et al. Eco-reliable operation based on clean environmental condition for the grid-connected renewable energy hubs with heat pump and hydrogen, thermal and compressed air storage systems. Sci Rep. 2025;15(1):464. doi: 10.1038/s41598-024-84231-4 39747608 PMC11696766

[38] Koumparakis C, Kountouris I, Bramstoft R. Utilization of excess heat in future Power-to-X energy hubs through sector-coupling. Appl Energy. 2025;377:124098. doi: 10.1016/j.apenergy.2024.124098

[39] Wang L, Su C, Liang B, Feng C, Zhang Y. Security constrained optimal power system dispatch considering stochastic power facility failures under extreme precipitation. Electric Power Syst Res. 2025;239:111214. doi: 10.1016/j.epsr.2024.111214

[40] Abdulnasser G, Ali A, Shaaban MF, Mohamed EEM. Optimal resource allocation and operation for smart energy hubs considering hydrogen storage systems and electric vehicles. Energy. 2024;295:130826. doi: 10.1016/j.energy.2024.130826

[41] Hu Z, Su R, Veerasamy V, Huang L, Ma R. Resilient frequency regulation for microgrids under phasor measurement unit faults and communication intermittency. IEEE Trans Ind Informat. 2024;21(2):1941–49.

[42] Li X, Hu C, Luo S, Lu H, Piao Z, Jing L. Distributed hybrid-triggered observer-based secondary control of multi-bus DC microgrids over directed networks. IEEE Trans Circuits Syst I Regul Pap. 2025;72(5):2467–80.

[43] Shokri M, Niknam T, Mohammadi M, Dehghani M, Siano P, Ouahada K, et al. A novel stochastic framework for optimal scheduling of smart cities as an energy hub. IET Generation Trans Dist. 2024;18(14):2421–34. doi: 10.1049/gtd2.13202

[44] Jadidbonab M, Dolatabadi A, Mohammadi-Ivatloo B, Abapour M, Asadi S. Risk-constrained energy management of PV integrated smart energy hub in the presence of demand response program and compressed air energy storage. IET Renewable Power Gen. 2019;13(6):998–1008. doi: 10.1049/iet-rpg.2018.6018

[45] Imanloozadeh A, Nazififard M, Sadat SA. A new stochastic optimal smart residential energy hub management system for desert environment. Int J Energy Res. 2021;45(13):18957–80. doi: 10.1002/er.6991

[46] Dolatabadi A, Jadidbonab M, Mohammadi-ivatloo B. Short-term scheduling strategy for wind-based energy hub: a hybrid stochastic/IGDT approach. IEEE Trans Sustain Energy. 2019;10(1):438–48. doi: 10.1109/tste.2017.2788086

[47] Majidi M, Zare K. Integration of smart energy hubs in distribution networks under uncertainties and demand response concept. IEEE Trans Power Syst. 2019;34(1):566–74. doi: 10.1109/tpwrs.2018.2867648

[48] Ostovar S, Moeini-Aghtaie M, Hadi MB. Flexibility provision of residential energy hubs with demand response applications. IET Gener Trans Dist. 2022;16(8):1668–79. doi: 10.1049/gtd2.12392

[49] Nosratabadi SM, Jahandide M, Guerrero JM. Robust scenario-based concept for stochastic energy management of an energy hub contains intelligent parking lot considering convexity principle of CHP nonlinear model with triple operational zones. Sustain Cities Soc. 2021;68:102795. doi: 10.1016/j.scs.2021.102795

[50] Rakipour D, Barati H. Probabilistic optimization in operation of energy hub with participation of renewable energy resources and demand response. Energy. 2019;173:384–99. doi: 10.1016/j.energy.2019.02.021

[51] Palani M, Vedavalli M, Veeramani V, Sridharan S. Optimal operation of residential energy hubs include hybrid electric vehicle & heat storage system by considering uncertainties of electricity price and renewable energy. Energy. 2022;:124952.

[52] Moeini-Aghtaie M, Dehghanian P, Davoudi M. Energy management of plug-in hybrid electric vehicles in renewable-based energy hubs. Sustain Energy Grids Netw. 2022;32:100932. doi: 10.1016/j.segan.2022.100932

[53] Moghaddas-Tafreshi SM, Jafari M, Mohseni S, Kelly S. Optimal operation of an energy hub considering the uncertainty associated with the power consumption of plug-in hybrid electric vehicles using information gap decision theory. Int J Electr Power Energy Syst. 2019;112:92–108. doi: 10.1016/j.ijepes.2019.04.040

[54] Emrani-Rahaghi P, Hashemi-Dezaki H, Hasankhani A. Optimal stochastic operation of residential energy hubs based on plug-in hybrid electric vehicle uncertainties using two-point estimation method. Sustain Cities Soc. 2021;72:103059.

[55] Yang C, Wu Z, Li X, Fars A. Risk-constrained stochastic scheduling for energy hub: integrating renewables, demand response, and electric vehicles. Energy. 2024;288:129680.

[56] Anastasiadis AG, Lekidis A, Pierros I, Polyzakis A, Vokas GA, Papageorgiou EI. Energy cost optimization for incorporating energy hubs into a smart microgrid with RESs, CHP, and EVs. Energies. 2024;17(12):2827. doi: 10.3390/en17122827

[57] Yan L, Deng X, Li J. Integrated energy hub optimization in microgrids: Uncertainty-aware modeling and efficient operation. Energy. 2024;291:130391. doi: 10.1016/j.energy.2024.130391

[58] Wang G, Zhou Y, Lin Z, Zhu S, Qiu R, Chen Y, et al. Robust energy management through aggregation of flexible resources in multi-home micro energy hub. Appl Energy. 2024;357:122471. doi: 10.1016/j.apenergy.2023.122471

[59] Norouzi S, Mirzaei MA, Zare K, Shafie-khah M, Nazari-Heris M. A second-order stochastic dominance-based risk-averse strategy for self-scheduling of a virtual energy hub in multiple energy markets. IEEE Access. 2024;12:84333–84351.

[60] Huang Z, Xu L, Wang B, Li J. Optimizing power systems and microgrids: a novel multi-objective model for energy hubs with innovative algorithmic optimization. Int J Hydrogen Energy. 2024;69:927–43. doi: 10.1016/j.ijhydene.2024.04.341

[61] Kioumarsi K, Bolurian A. Optimal energy management for electric vehicle charging parking lots with considering renewable energy resources and accurate battery characteristic modeling. J Energy Storage. 2025;107:114914. doi: 10.1016/j.est.2024.114914

[62] Kumar A, Maulik A, Chinmaya KA. A decentralized energy management scheme for a DC microgrid with correlated uncertainties and integrated demand response. Electric Power Syst Res. 2025;238:111093. doi: 10.1016/j.epsr.2024.111093

[63] Aghdam FH, Mohammadi-ivatloo B, Abapour M, Marzband M, Rasti M, Pongracz E. Enhancing the risk-oriented participation of wind power plants in day-ahead, balancing, and hydrogen markets with shared multi-energy storage systems. J Energy Storage. 2025;107:114911. doi: 10.1016/j.est.2024.114911

[64] Zheng J, Liang Z-T, Li Y, Li Z, Wu Q-H. Multi-agent reinforcement learning with privacy preservation for continuous double auction-based P2P energy trading. IEEE Trans Ind Inf. 2024;20(4):6582–90. doi: 10.1109/tii.2023.3348823

[65] Qais M, Kirli D, Moroshko E, Kiprakis A, Tsaftaris S. A virtual power plant for coordinating batteries and EVs of distributed zero-energy houses considering the distribution system constraints. J Energy Storage. 2025;106:114905. doi: 10.1016/j.est.2024.114905

[66] Fu Y, Dong M, Zhou L, Li C, Yu FR, Cheng N. A distributed incentive mechanism to balance demand and communication overhead for multiple federated learning tasks in IoV. IEEE Internet Things J. 2025;12(8):10479–92.

[67] Ozkaya B, Duman S, Kahraman HT, Guvenc U. Optimal solution of the combined heat and power economic dispatch problem by adaptive fitness-distance balance based artificial rabbits optimization algorithm. Expert Syst Appl. 2024;238:122272. doi: 10.1016/j.eswa.2023.122272

[68] Sharifzadeh H. Two efficient logarithmic formulations to solve nonconvex economic dispatch. Electric Power Syst Res. 2024;229:110123. doi: 10.1016/j.epsr.2024.110123

[69] Xia Y, Huang Y, Fang J. A generalized Nash-in-Nash bargaining solution to allocating energy loss and network usage cost of buildings in peer-to-peer trading market. Sustain Energy Grids Netw. 2025;42:101628.

[70] Khalid M. A techno-economic framework for optimizing multi-area power dispatch in microgrids with tie-line constraints. Renew Energy. 2024;231:120854. doi: 10.1016/j.renene.2024.120854

[71] Ahmed I, Irshad A, Zafar S, Khan BA, Raza M, Ali PR. The role of environmental initiatives and green value co-creation as mediators: promoting corporate entrepreneurship and green innovation. SN Bus Econ. 2023;3(4):85. doi: 10.1007/s43546-023-00465-w

[72] Ahmed I, Alvi A, Ashfaq B, Mukhtar S, Ali PR. Technological, financial and ecological analysis of photovoltaic power system using retscreen: A case in Khuzdar, Pakistan. In: 2022 International Conference on Emerging Technologies in Electronics, Computing and Communication (ICETECC). IEEE; 2022, pp. 1–6.

[73] Zhao H, Zong Q, Zhou H, Yao W, Sun K, Zhou Y, et al. Frequency-voltage active support strategy for hybrid wind farms based on grid-following and grid-forming hierarchical subgroup control. CSEE J Power Energy Syst. 2025;11(1):65–77.

[74] Najafi A, Tavakoli A, Pourakbari-Kasmaei M, Lehtonen M. A risk-based optimal self-scheduling of smart energy hub in the day-ahead and regulation markets. J Clean Prod. 2021;279:123631. doi: 10.1016/j.jclepro.2020.123631

[75] Zhang X, Che L, Shahidehpour M, Alabdulwahab AS, Abusorrah A. Reliability-based optimal planning of electricity and natural gas interconnections for multiple energy hubs. IEEE Trans Smart Grid. 2015;8(4):1658–67.

[76] Qamar N, Malik TN, Qamar F, Ali M, Naeem M. Energy hub: modeling, control, and optimization. In: Renew Energy Systems. Academic Press; 2021, pp. 339–62.

[77] Javadi MS, Anvari-Moghaddam A, Guerrero JM, Nezhad E, Lotfi M, Catalão JP. Optimal operation of an energy hub in the presence of uncertainties. In: 2019 IEEE International Conference on Environment and Electrical Engineering and 2019 IEEE Industrial and Commercial Power Systems Europe (EEEIC/I&CPS Europe). IEEE; 2019.

[78] Rastegar M, Fotuhi-Firuzabad M, Zareipour H, Moeini-Aghtaieh M. A probabilistic energy management scheme for renewable-based residential energy hubs. IEEE Trans Smart Grid. 2017;8(5):2217–27. doi: 10.1109/tsg.2016.2518920

[79] Tavakoli A, Karimi A, Shafie-khah M. Optimal probabilistic operation of energy hub with various energy converters and electrical storage based on electricity, heat, natural gas, and biomass by proposing innovative uncertainty modeling methods. J Energy Storage. 2022;51:104344. doi: 10.1016/j.est.2022.104344

[80] Qamar N, Nadeem Malik T. Smart energy hub optimization in presence of stochastically modeled renewables and loads. Int J Energy Res. 2021;46(3):2858–75. doi: 10.1002/er.7349

[81] Wu X, Hu X, Yin X, Moura SJ. Stochastic optimal energy management of smart home with PEV energy storage. IEEE Trans Smart Grid. 2018;9(3):2065–75. doi: 10.1109/tsg.2016.2606442

[82] Sadeghi H, Rashidinejad M, Moeini-Aghtaie M, Abdollahi A. The energy hub: an extensive survey on the state-of-the-art. Appl Therm Eng. 2019;161:114071.

[83] Nixon T, Curry RM, Allaissem B. P. Mixed-integer programming models and heuristic algorithms for the maximum value dynamic network flow scheduling problem. Comput Oper Res. 2025;175:106897. doi: 10.1016/j.cor.2024.106897

[84] Güngör M. Classification and comparison of integer programming formulations for the single-machine sequencing problem. Comput Oper Res. 2025;173:106844. doi: 10.1016/j.cor.2024.106844

[85] Llorens-Iborra F, Riquelme-Santos J, Romero-Ramos E. Mixed-integer linear programming model for solving reconfiguration problems in large-scale distribution systems. Electric Power Syst Res. 2012;88:137–45. doi: 10.1016/j.epsr.2012.01.014

[86] Montoya OD, Raez-Vanegas CA, González-Granada JR. Dynamic active and reactive power compensation in distribution networks using PV-STATCOMs: a tutorial using the Julia software. Results Eng. 2024:101876.

[87] Kasani AA, Esmaeili A, Golzary A. Software tools for microalgae biorefineries: cultivation, separation, conversion process integration, modeling, and optimization. Algal Res. 2022;61:102597.

[88] Emrani-Rahaghi P, Hashemi-Dezaki H. Optimal scenario-based operation and scheduling of residential energy hubs including plug-in hybrid electric vehicle and heat storage system considering the uncertainties of electricity price and renewable distributed generations. J Energy Storage. 2021;33:102038. doi: 10.1016/j.est.2020.102038

