# Expression of Concern: Stochastic optimization for minimizing operational costs in smart hybrid energy networks considering electric vehicle

**DOI:** 10.1371/journal.pone.0357060

**Published:** 2026-08-27

**Authors:** The PLOS One Editors

After this article [1] was published, the following concerns were noted:

- The articles cited as References 7, 15, 18, 20, 21, 24, 38, 39, and 64 appear to be irrelevant to this article [1].
- The article cited as Reference 37 was retracted after [1] was published.

In addition, the *PLOS One* Editors have concerns about the article’s compliance with the PLOS Authorship policy.

We regret that the issues were not identified prior to the article’s publication.

In light of the cumulative issues, the *PLOS One* Editors issue this Expression of Concern. Readers are advised to interpret the article [1] with caution.

## References

[1] Qamar N, Alqahtani M, Rehan M, Ahmed I, Khalid M. Stochastic optimization for minimizing operational costs in smart hybrid energy networks considering electric vehicle. PLoS One. 2025;20(6):e0323491. doi: 10.1371/journal.pone.0323491 40489454 PMC12148165

