# Supplementary material for: Stochastic optimization for minimizing operational costs in smart hybrid energy networks considering electric vehicle
Source: PLoS One. 2025 Jun 9;20(6):e0323491. doi: 10.1371/journal.pone.0323491 (PMC12148165; doi:10.1371/journal.pone.0323491)
Supplement: Attachment — Submitted filename: Response.pdf [file pone.0323491.s001.pdf]

Manuscript Number: PONE-D-25-06050
Original Article Title: "Stochastic Optimization for Minimizing Operational Costs in Smart Hybrid Energy Networks Considering Electric Vehicle"

Response to Comments from Academic Editor (PLOS ONE)

Comment: **Thank you for submitting your manuscript to PLOS ONE. After careful consideration, we feel that it has merit but does not fully meet PLOS ONE's publication criteria as it currently stands. Therefore, we invite you to submit a revised version of the manuscript that addresses the points raised during the review process.**
Please submit your revised manuscript by Apr 24 2025 11:59PM. If you will need more time than this to complete your revisions, please reply to this message or contact the journal office at plosone@plos.org. When you're ready to submit your revision, log on to https://www.editorialmanager.com/pone/ and select the 'Submissions Needing Revision' folder to locate your manuscript file. Please include the following items when submitting your revised manuscript:

1. **A rebuttal letter that responds to each point raised by the academic editor and reviewer(s). You should upload this letter as a separate file labeled 'Response to Reviewers'.**
2. **A marked-up copy of your manuscript that highlights changes made to the original version. You should upload this as a separate file labeled 'Revised Manuscript with Track Changes'.**
3. **An unmarked version of your revised paper without tracked changes. You should upload this as a separate file labeled 'Manuscript'**

Response: We appreciate the time and effort the reviewers and the Editor-in-Chief have dedicated to evaluating the manuscript with Manuscript Ref. No.: PONE-D-25-06050 titled **"Stochastic Optimization for Minimizing Operational Costs in Smart Hybrid Energy Networks Considering Electric Vehicle"**. We also appreciate the reviewers feedback and am willing to revise the manuscript based on their suggestions. We have addressed all comments (highlighted in RED) raised by the reviewers. Please refer to my specific responses regarding the suggested improvements in the manuscript. Thank you for providing the opportunity to revise my manuscript.

Manuscript Number: PONE-D-25-06050
Original Article Title:"Stochastic Optimization for Minimizing Operational Costs in Smart Hybrid Energy Networks Considering Electric Vehicle"

Response to Comments from Reviewer 1

Overall Comment: **This paper proposes a data-driven distributionally robust optimization approach for integrated energy systems. While the paper is well-written, I still have the following comment:**

Response: We sincerely appreciate the reviewer's positive feedback on our manuscript and their valuable comments, which have helped us improve the quality of our work. We have carefully considered all the reviewer's suggestions and have thoroughly revised the manuscript accordingly. To ensure transparency, all modifications have been highlighted in red text within the revised manuscript.

Comment 1: **It is recommended to delete the second column of the table, as all the literature includes RES, making it non-comparative.**

Response: We appreciate the reviewer's insightful suggestion. As per the recommendation, we have revised Table 1 by removing the second column to enhance its comparative value. The changes have been highlighted in red text on page 3 of the revised manuscript (also on page 3 in response file). We believe this revision improves the clarity and relevance of the table.

Comment 2: **The studied REH structure is relatively simple. It is suggested to extend the components or provide more details about the component models, such as modeling different types of transferable and non-transferable loads separately.**

Response: We appreciate the reviewer's valuable suggestion. As per the recommendation, we have added a brief paragraph providing more details about the component models, including the modeling of different types of transferable and non-transferable loads separately. The revisions are highlighted in red text on pages 6 and 7 of the revised manuscript.
The following paragraph has been incorporated:

Table 1: Comparison of the proposed SREH with relevant literature

References	Uncertainty modeling of RES	DSM	PHEV	Uncertainty modeling of PHEV	Smart charging of PHEV	
44	✓	✓	✓	×	×	×
45	✓	✓	✓	×	×	×
40	✓	✓	✓	✓	×	×
46	✓	✓	×	×	×	×
47	✓	✓	✓	×	×	×
48	✓	✓	✓	✓	×	×
49	✓	✓	×	✓	✓	×
50	✓	✓	✓	×	×	×
51	✓	✓	✓	✓	×	×
52	✓	×	×	✓	✓	✓
53	✓	×	×	✓	✓	✓
54	✓	✓	×	✓	✓	×
55	✓	✓	✓	✓	×	×
56	✓	×	×	✓	×	×
57	✓	✓	✓	✓	×	×
58	✓	✓	✓	×	×	×
59	✓	✓	✓	✓	✓	×
60	✓	✓	✓	×	×	×
Proposed Model	✓	✓	✓	✓	✓	✓

The REH includes various components, such as CHP, which converts gas energy into heat and electrical energy; RES, which produces electrical energy; electric chillers and absorption chillers that fulfill cooling demand; and a boiler that generates heat using natural gas. To better fulfill REH's load demand, electrical loads are further divided into two types: shiftable and non-shiftable. Shiftable loads are flexible and can operate at any time of the day. These include appliances such as dishwashers, washing machines, and water pumps. On the other hand, one must operate non-shiftable loads as needed, without the flexibility to shift them to a different time of day. These include household appliances such as lighting and heating/cooling systems.

Comment 3: **It is advisable to list and analyze the results for each case, as this would make the comparisons clearer and easier to understand.**

Response: We appreciate the reviewer's insightful suggestion. As per the recommendation, we have thoroughly revised the manuscript by incorporating two new subsections, "**Cases Summary**" and "**Discussion**," on pages 23 and 24, with changes highlighted in red text. Additionally, we have added a new table (Table 2 in response file and Table 8 in manuscript) presenting a comparative **Operational Cost Analysis of SREH under Different Case Studies** to enhance clarity and understanding.
The following text has been incorporated:

Cases Summary

The first case is a base case in which the total operational cost of REH is 7675.00 USD. The second case incorporates the PHEV without any smart charging mechanism, which increases the operational cost of REH by 0.63% due to the additional charging of PHEV. The third case involves the smart charging mechanism, which takes care of peak/off-peak hours and smartly charges/discharges the PHEV. The employed smart charging mechanism decreases the cost of SREH by 1.98% while also charging the PHEV and increases the reliability of REH as well. The fourth case involves DSM in addition to smart charging, which results in a further decrease in cost by 3.06%. A summary of these results is shown in Table 8.

Table 2: Operational Cost Analysis of SREH under Different Case Studies

Case Study	PHEV Inclusion	Smart Charging of PHEV	Demand Side Management	Total Cost (USD)	% Change
I	-	-	-	7675.00	-
II	✓	-	-	7723.70	+0.63
III	✓	✓	-	7523.10	-1.98
IV	✓	✓	✓	7439.68	-3.06

Discussion

The first case is the base case, containing all the basic components of the REH, such as RES, CHP, AC, EC, a boiler, and electrical, heating, and cooling loads, but without the involvement of a PHEV. After a proper sensitivity analysis of key REH parameters, the sizes of different components are carefully selected, keeping practical systems in mind. The parameters of RES and central devices are optimally selected so that they are neither too large, which would result in high investment or operational costs, nor too small, making their impact negligible. After selecting the desired configuration, the REH is optimized for its operational cost over 24 hours. The second case involves incorporating a PHEV into the REH without any smart charging mechanism to analyze the impact of adding a PHEV to the REH. The unmanaged charging of the PHEV increases the operational cost of the REH, highlighting the need for proper smart charging of the PHEV. In the third case, a smart charging mechanism is introduced that optimally charges/discharges the PHEV, taking into account the pricing of purchased electricity at any given time. The PHEV charging/discharging process is specified as a set of decision variables based on the pricing mechanism. This enables the PHEV to be charged when electricity prices are low and discharged when they are high. The flexibility of discharging the PHEV to obtain electrical energy when the system is under stress increases the stability and reliability of the REH, making it more robust

and resilient. The fourth case involves DSM, which shifts some portion of the load when required. When there is increased load stress on the REH during peak hours, DSM makes it possible to optimally shift some shiftable loads to off-peak hours, reducing the need to purchase high-cost energy. This strategy not only increases the stability of the REH but also decreases its operational cost.

Comment 4: **It is recommended to expand the conclusion section to outline the limitations of this study and provide a more detailed discussion of future research needs.**

Response: We appreciate the reviewer's valuable suggestion. As per the recommendation, we have fully revised the Conclusion section by adding the limitations of this study and providing a more detailed discussion of future research needs. The revised Conclusion is highlighted in red text in the manuscript.
The following revision has been incorporated.
This article presents a stochastic MILP model of REH that integrates with RES in the presence of PHEV. Four case studies were conducted to assess the effectiveness of the proposed scheme. Firstly, the proposed scheme optimizes REH in the presence of RES, meeting its electrical demand through a combination of CHP, RES, and grid-purchased electricity. The results show that when the price of gas is less than the electricity price, the CHP unit generates more electrical energy from the gas to avoid the costly purchase of electricity. EHP converts the electrical energy into cooling power instead of heating energy due to the high demand for cooling energy in summer weather. Without any charging management, adding the PHEV to the REH setup causes the operating cost of REH to increase, rather than decrease, as it charges during peak hours. However, smart charging and discharging of the PHEV allow SREH to significantly reduce its operational expenses. Smart management of a PHEV battery enables the consumption of stored electrical energy during peak hours, eliminating the need to purchase it from the grid and recharging it during off-peak hours for the next day's trip. Finally, the DSM technique is applied to all types of loads to further reduce operational costs without compromising customer comfort. In terms of practical applications, the proposed model can be implemented in smart buildings, residential apartments, and societies to optimize energy efficiency and reduce reliance on expensive grid electricity. By implementing smart energy management strategies, real-world applications of this model can significantly reduce energy costs and carbon footprints for residential consumers. To make the overall energy infrastructure more

resilient and cost-effective, urban planners and policymakers can apply these insights to build sustainable microgrids that seamlessly integrate RES, PHEVs, and ESS. This study has not considered the actual degradation of batteries in PHEVs and their long-term economic implications. Future research should aim to incorporate the impact of battery degradation and its replacement costs. Investigating the integration of emerging technologies, such as hydrogen storage and vehicle-to-grid interactions, could further improve the flexibility and sustainability of the REH.

Comment 5: **The proposed optimization method has already been extensively researched, thus the contribution of the manuscript is insufficient. It is suggested to highlight the novelty of the proposed method and conduct a comparative analysis with existing methods.**

Response: We appreciate the reviewer's feedback. To address this concern, we have explicitly highlighted the novelty of the proposed method and conducted a comparative analysis with existing methods in **Subsection 1.1 (Contribution and Novelties)**. Furthermore, we have added a paragraph summarizing the comparative analysis. All revisions are highlighted in red text in the manuscript.
The following revision has been made:

Contribution and Novelties

The following outline highlights the key contributions of this research to the field of optimal stochastic operation of REH.

1. In contrast to earlier research in [61-64], a more complex and practical model has been developed that takes into account the uncertain charging limits of both RES and PHEVs, as well as factors like arrival time, travel distance, smart charging/discharging, and departure time. The developed model is more reliable and achieved optimal economic aspects in terms of cost and reliability.
2. The proposed approach signifies a notable improvement over prior research works by concurrently tackling the uncertainties linked to RES and PHEVs, resulting in enhanced accuracy and reliability of outcomes. The simultaneous examination of uncertainty improves the model's robustness and complexity, distinguishing it from previous models that addressed either RES [40,56] or PHEV challenges [52,65] individually.

3. The suggested methodology is novel relative to previous works [17,23,66], since it incorporates an intelligent decision-making process for the charging and discharging of PHEVs into the REH scheduling to enhance its optimal performance.

4. This study presents a broader strategy by concurrently applying DSM approaches to electrical, heating, and cooling loads, in contrast to prior studies such as [67,68], which concentrated exclusively on dispatch models for electric loads. The incorporation of heating and cooling systems substantially improves the model's relevance, making it more adept at handling the intricate and varied load requirements in contemporary energy systems.

5. The proposed work conducted a comparative study of all contributions (see Table 1 references [32-48]) and [69] to ascertain the relative efficacy of each comparative feature (DSM, smart charging of PHEVs, and the unpredictable nature of RES).

6. Unlike the works in [9,20,70], which use distributed methods to combine RES through RETScreen, the proposed framework is based on MILP and GAMS software, which gives better outcomes and is simple to use for complex energy delivery problems.

The proposed method introduces significant improvements over existing methods, such as smart charging strategies for PHEVs that dynamically adjust based on real-time demand, electricity prices, and the availability of RES. This feature enhances the efficiency of the SREH by optimizing the charging/discharging cycles of PHEVs in addition to the DSM. The combined stochastic modeling of solar irradiance, wind speed, and PHEV usage patterns provides more accurate forecasting and optimization under real-world uncertainties, which is crucial for optimizing EHs in smart grids. Another novel aspect of our technique is the identification of the feasible operating region for the SREH components based on their constraints. Previous methods mainly focus on the isolated optimization of PHEV charging/discharging and RES without considering real-time interactions between them. The proposed method improves on this approach by simultaneously optimizing the charging/discharging behavior of PHEVs and the energy management of the REH while also incorporating DSM strategies. This results in a more comprehensive and adaptive solution for energy management in modern grids.

Comment 6: **What are the differences between the proposed intelligent charging and discharging strategy for PHEVs and that of a regular battery?**

Response: We appreciate the reviewer's insightful query. To address this, we have added a detailed explanation of the differences between the proposed intelligent charging/discharging strategy for PHEVs and that of a regular battery. Please see the revised text highlighted in red in Section 3.7: Last paragraph.

The following explanation has been incorporated:

The difference between a regular battery and a PHEV battery is that a regular battery is available all day, making it easier to model. In contrast, a PHEV battery is only available when the vehicle is at the building. Additionally, a PHEV battery discharges during travel, whereas a conventional battery only discharges to meet the demands of the REH during peak hours. There are also additional constraints associated with a PHEV battery, such as the need for it to be fully charged before departure time to ensure the comfort level of the driver. These additional constraints need to be managed effectively by an intelligent charging mechanism.

-Thank you very much-

Manuscript Number: PONE-D-25-06050
Original Article Title: "Stochastic Optimization for Minimizing Operational Costs in Smart Hybrid Energy Networks Considering Electric Vehicle"

Response to Comments from Reviewer 2

Overall Comment **This paper focuses on the optimization of the operational costs of Residential Energy Hubs (REHs) in smart hybrid energy networks. The proposed stochastic model and related strategies demonstrate certain innovativeness. However, there are some areas that need to be improved, and my comments are as follows:**

Response: Thank you for your constructive feedback. We have revised the manuscript according to your comments to improve clarity and rigor. The revised manuscript, with all changes highlighted in red text, is now more aligned with your suggestions.

Comment 1: **The assumptions for handling the uncertainties of Renewable Energy Sources (RES) and Plug - in Hybrid Electric Vehicles (PHEVs) in the paper are relatively simplistic. It is advisable to introduce more complex stochastic processes or probability distribution models to enhance the accuracy and practicality of the model.**

Response: We appreciate the reviewer's valuable suggestion. To enhance the accuracy and practicality of the model, **Weibull probability distribution models** were used for handling the uncertainties of RES and PHEVs (Figure 1 in response file). To better clarify this, we have revised the manuscript, and the changes are highlighted in red text. Please see **Section 2.2:** First paragraph for details.
The following revision has been made:
In this article, probability density functions (PDFs) are used to model the random behavior of PHEVs, wind speed, and solar irradiance. The normal PDF is used to model uncertainties related to PHEVs, the Weibull PDF is used for wind speed, and the beta PDF is used to model solar irradiance. The powers generated from solar and wind are calculated using (3) and (4), respectively.

$$P^{e}_{\mathrm{PV}}(S_i(t,s)) = \mathrm{PV}_{\mathrm{array}} \cdot \mathrm{df}_{\mathrm{PV}} \cdot S_{i,\mathrm{STC}} \cdot (1 + \alpha_p\,(T_c - T_{c,\mathrm{STC}})) \tag{3}$$

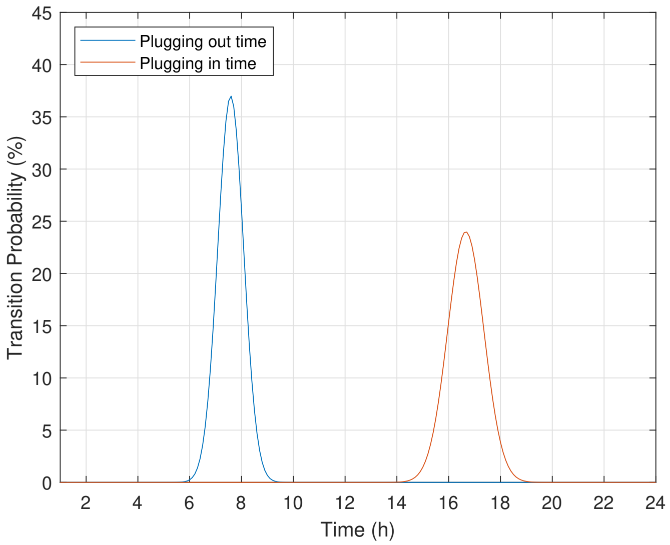

Figure 1: Probability of PHEV's time of departure and time of arrival.

where PV_{array} is the size of PV panels, df_{PV} is the derating factor of PV panels, $S_{i,\text{STC}}$ is the solar irradiance at standard test conditions, α_p is the coefficient of temperature, and $T_{c,\text{STC}}$ is the PV cell temperature under standard test conditions.

$$P^e_{\text{wind}}(\omega(t,s)) = \begin{cases} P^w_{\text{out}} & \text{if } \omega^w_r \leq \omega_t \leq \omega_{\text{ocut}} \\ \frac{P^w_{\text{out}}(\omega_t - \omega^{\text{cut}}_i)}{(\omega^w_r - \omega^{\text{cut}}_i)} & \text{if } \omega^{\text{cut}}_i \leq \omega_t \leq \omega^w_r \\ 0 & \text{if } \omega_t \leq \omega^{\text{cut}}_i \text{ or } \omega_t \geq \omega_{\text{ocut}} \end{cases} \tag{4}$$

where P^w_{out} is the power output of wind turbine (WT), ω^w_r is the rated speed of WT, ω^{cut}_i is the cut-in speed of WT, and ω^{cut}_o is the cut-out speed of WT.

Comment 2: **Sensitivity analysis should be added to clarify the impact degree of each parameter on the results, providing more targeted guidance for the optimization of practical systems.**

Response: We appreciate the reviewer's valuable suggestion. As per the recommendation, a detailed **"Discussion"** section has been added to clarify the impact degree of each parameter on the results, providing more targeted guidance for the optimization of practical systems. Please see the revised version highlighted in red text on page 23.
The following revision has been made:

Discussion

The first case is the base case, containing all the basic components of the REH, such as RES, CHP, AC, EC, a boiler, and electrical, heating, and cooling loads, but without the involvement of a PHEV. After a proper sensitivity analysis of key REH parameters, the sizes of different components are carefully selected, keeping practical systems in mind. The parameters of RES and central devices are optimally selected so that they are neither too large, which would result in high investment or operational costs, nor too small, making their impact negligible. After selecting the desired configuration, the REH is optimized for its operational cost over 24 hours. The second case involves incorporating a PHEV into the REH without any smart charging mechanism to analyze the impact of adding a PHEV to the REH. The unmanaged charging of the PHEV increases the operational cost of the REH, highlighting the need for proper smart charging of the PHEV. In the third case, a smart charging mechanism is introduced that optimally charges/discharges the PHEV, taking into account the pricing of purchased electricity at any given time. The PHEV charging/discharging process is specified as a set of decision variables based on the pricing mechanism. This enables the PHEV to be charged when electricity prices are low and discharged when they are high. The flexibility of discharging the PHEV to obtain electrical energy when the system is under stress increases the stability and reliability of the REH, making it more robust and resilient. The fourth case involves DSM, which shifts some portion of the load when required. When there is increased load stress on the REH during peak hours, DSM makes it possible to optimally shift some shiftable loads to off-peak hours, reducing the need to purchase high-cost energy. This strategy not only increases the stability of the REH but also decreases its operational cost.

Comment 3: **Although the four case studies compare the operation of REHs under different scenarios, the experimental design is not comprehensive enough. It is recommended to add case analyses of different seasons and regions to make the research results more universal.**

Response: We appreciate the reviewer's insightful suggestion. As per the recommendation, we have added a "**Cases Summary**" subsection on page 23 to provide a detailed analysis of each case. Additionally, Table 8 has been incorporated to clarify the cost analysis. Please see the revised version highlighted in red text. The following text has been incorporated:

Cases Summary

The first case is a base case in which the total operational cost of REH is 7675.00 USD. The second case incorporates the PHEV without any smart charging mechanism, which increases the operational cost of REH by 0.63% due to the additional charging of PHEV. The third case involves the smart charging mechanism, which takes care of peak/off-peak hours and smartly charges/discharges the PHEV. The employed smart charging mechanism decreases the cost of SREH by 1.98% while also charging the PHEV and increases the reliability of REH as well. The fourth case involves DSM in addition to smart charging, which results in a further decrease in cost by 3.06%. A summary of these results is shown in Table 8 (Table 1 in response file).

Comment 4:**The analysis of the experimental results is rather superficial, mainly staying at the level of data comparison. It is necessary to deeply explore the reasons behind the data, such as the specific mechanisms by which the smart charging mechanism and the Demand Response Program (DRP) reduce costs, as well as their impacts on the stability and reliability of the energy system.**

Response: We appreciate the reviewer's valuable feedback. As per the recommendation, we have provided a detailed analysis of the experimental results, emphasizing the impact of the smart charging mechanism and Demand Side Management (DSM) on the stability and reliability of the energy system. Please refer to the **Discussion section** in the response file on page 11 for details.
The following text has been added:
In the third case, a smart charging mechanism is introduced that optimally charges/discharges the PHEV, taking into account the pricing of purchased electricity at any given time. The PHEV charging/discharging process is specified as a set of decision variables based on the pricing mechanism. This enables the PHEV to be charged when electricity prices are low and discharged when they are high. The flexibility of discharging the PHEV to obtain electrical energy when the system is under stress increases the stability and reliability of the REH, making it more robust and resilient. The fourth case involves DSM, which shifts some portion of the load when required. When there is increased load stress on the REH during peak hours, DSM makes it possible to optimally shift some shiftable loads to off-peak hours, reducing the need to purchase high-cost energy. This strategy not only increases the stability of the REH but also decreases its operational cost.

Comment 5: **The research results are not closely integrated with practical applications. It is recommended to discuss this in the conclusion.**

Response: We appreciate the reviewer's insightful suggestion. As per the recommendation, we have revised the Conclusion section to discuss the practical applications of our research along with future research directions. Please see the revised version highlighted in red text.

The following revision has been made:

This article presents a stochastic MILP model of REH that integrates with RES in the presence of PHEV. Four case studies were conducted to assess the effectiveness of the proposed scheme. Firstly, the proposed scheme optimizes REH in the presence of RES, meeting its electrical demand through a combination of CHP, RES, and grid-purchased electricity. The results show that when the price of gas is less than the electricity price, the CHP unit generates more electrical energy from the gas to avoid the costly purchase of electricity. EHP converts the electrical energy into cooling power instead of heating energy due to the high demand for cooling energy in summer weather. Without any charging management, adding the PHEV to the REH setup causes the operating cost of REH to increase, rather than decrease, as it charges during peak hours. However, smart charging and discharging of the PHEV allow SREH to significantly reduce its operational expenses. Smart management of a PHEV battery enables the consumption of stored electrical energy during peak hours, eliminating the need to purchase it from the grid and recharging it during off-peak hours for the next day's trip. Finally, the DSM technique is applied to all types of loads to further reduce operational costs without compromising customer comfort. In terms of practical applications, the proposed model can be implemented in smart buildings, residential apartments, and societies to optimize energy efficiency and reduce reliance on expensive grid electricity. By implementing smart energy management strategies, real-world applications of this model can significantly reduce energy costs and carbon footprints for residential consumers. To make the overall energy infrastructure more resilient and cost-effective, urban planners and policymakers can apply these insights to build sustainable microgrids that seamlessly integrate RES, PHEVs, and ESS. This study has not considered the actual degradation of batteries in PHEVs and their long-term economic implications. Future research should aim to incorporate the impact of battery degradation and its replacement costs. Investigating the integration of emerging technologies, such as hydrogen storage and vehicle-

to-grid interactions, could further improve the flexibility and sustainability of the REH.

Comment 6: **It is advisable to update the literature review, supplement the latest research progress, clarify the relationship between this research and the cutting - edge achievements, and highlight the innovativeness and necessity of the research. It is recommended to supplement the following literature (not mine): 10.1109/TCSI.2024.3523339; 10.1109/TII.2024.3495785.**

Response: We appreciate the reviewer's valuable suggestion. As per the recommendation, we have updated the literature review by incorporating the latest research progress and clarifying the relationship between our study and cutting-edge achievements to highlight its innovativeness and necessity. The mentioned literature (10.1109/TCSI.2024.3523339; 10.1109/TII.2024.3495785) has been added to the revised manuscript and highlighted in red text. Please see References 41 and 42.

The following text has been incorporated:

Some authors have applied the concept of a microgrid as a decentralized EH by integrating and managing different energy sources. Their dynamic model of control systems enables efficient, sustainable, and resilient energy management, allowing it to operate independently from the main grid while providing reliable power [41,42].

-Thank you very much-
